# Supplementary material for: Respiratory pathology in the mdx/utrn -/- mouse: A murine model for Duchenne Muscular Dystrophy (DMD)
Source: PLoS One. 2025 Feb 7;20(2):e0316295. doi: 10.1371/journal.pone.0316295 (PMC11805407; doi:10.1371/journal.pone.0316295)
Supplement: S1 Data — (PDF) [file pone.0316295.s001.pdf]

Mouse	Genotype	Sex	Weight	Clasp 1	Clasp 2	Clasp Avg	Gait 1	Gait 2
G810	Mdx/utrn -/-	M	16.2	0	1	0.5	0	0
G1023	Mdx/utrn -/-	M	16.9	1.5	1.5	1.5	2	2
G1220	Mdx/utrn -/-	M	12.6	1	1	1	1	1
G1222	Mdx/utrn -/-	M	13.8	0	1	0.5	0	0

Mouse	Genotype	Sex	Weight	Clasp 1	Clasp 2	Clasp Avg	Gait 1	Gait 2
G998	WT	M	20.8	0	0	0	0	0
G1035	WT	M	23.3	0	0	0	0	0
G1036	WT	M	22.1	0	0	0	0	0
G1037	WT	M	21.9	0	0	0	0	0

Gait Avg	Kyph 1	Kyph2	Kyph Avg	Ledge 1	Ledge 2	Ledge Avg	Wire 1	Wire 2	Wire Avg
0	1	1	1	0	0	0	98	19	58.5
2	3	3	3	2	2	2	25	22	23.5
1	2	2	2	2	3	2.5	120	120	120
0	2	2	2	0	0	0	120	76	98

Gait Avg	Kyph 1	Kyph2	Kyph Avg	Ledge 1	Ledge 2	Ledge Avg	Wire 1	Wire 2	Wire Avg
0	0	0	0	0.5	0	0.25	120	120	120
0	0	0	0	0	0	0	120	120	120
0	0	0	0	0.5	0	0.25	120	120	120
0	0	0	0	0	0	0	120	120	120

Grip 1	Grip 2	Grip 3	Grip Avg
0.6	0.8	0.4	0.6
0.6	0.5	0.5	0.533333
1.1	0.9	0.8	0.933333
1.2	1.2	1	1.133333

Grip 1	Grip 2	Grip 3	Grip Avg
1.8	1.9	1.3	1.666667
1.8	1.5	1.7	1.666667
1.2	1.2	1.6	1.333333
1.6	1.6	1.2	1.466667

Mouse	Genotype	Sex	Weight	Clasp 1	Clasp 2	Clasp Avg	Gait 1	Gait 2	Gait Avg
G810	Mdx/utrn -	M	16.9	0	1	0.5	0	0	0
G1213	Mdx/utrn -	M	17.9	0	1	0.5	0	0	0
G1214	Mdx/utrn -	M	24.2	0	0	0	0	0	0
G1215	Mdx/utrn -	M	23.3	1	1	1	0	0	0
G1220	Mdx/utrn -	M	17.7	1	1	1	1	1	1
G1352	Mdx/utrn -	M	17	0	0	0	0	0	0

Mouse	Genotype	Sex	Weight	Clasp 1	Clasp 2	Clasp Avg	Gait 1	Gait 2	Gait Avg
G998	WT	M	22.2	0	0	0	0	0	0
G1037	WT	M	20.2	0	0	0	0	0	0
G1167	WT	M	27.2	0	0	0	0	0	0
G1168	WT	M	23.4	0	0	0	0	0	0
G1169	WT	M	24.1	0	0	0	0	0	0
G1368	WT	M	18.4	0	0	0	0	0	0

Kyph 1	Kyph2	Kyph Avg	Ledge 1	Ledge 2	Ledge Avg	Wire 1	Wire 2	Wire Avg	Grip 1
2	0	1	0	1	0.5	34	5	19.5	0.3
2	2	2	1	0	0.5	15	43	29	0.8
2	2	2	0	0.5	0.25	102	34	68	0.9
2	2	2	0	0	0	118	120	119	1.2
2	2	2	1	1	1	120	66	93	0.7
0	0	0	0	0	0	120	16	68	0.6

Kyph 1	Kyph2	Kyph Avg	Ledge 1	Ledge 2	Ledge Avg	Wire 1	Wire 2	Wire Avg	Grip 1
0	0	0	0	0.5	0.25	120	120	120	1.7
0	0	0	0	0	0	72	52	62	1.6
0	0	0	0	0	0	120	120	120	2
0	0	0	1	0	0.5	120	120	120	2.1
0	0	0	0	0	0	120	120	120	1.8
0	0	0	0	0	0	120	120	120	1.1

Grip 2	Grip 3	Grip Avg
0.9	0.9	0.7
0.7	0.7	0.733333
1	0.7	0.866667
0.8	0.9	0.966667
0.9	0.9	0.833333
0.8	0.9	0.766667

Grip 2	Grip 3	Grip Avg
2.6	1.3	1.866667
1.3	1.4	1.433333
2.1	1.8	1.966667
2.3	1.7	2.033333
1.9	2	1.9
1.4	1.6	1.366667

Mouse	Genotype	Sex	Weight	Clasp 1	Clasp 2	Clasp Avg	Gait 1	Gait 2	Gait Avg
G809	Mdx/utrn ·	M	18.9	0	1	0.5	0.5	0.5	0.5
G810	Mdx/utrn ·	M	17.7	0	1	0.5	0	0	0
G1213	Mdx/utrn ·	M	18.5	0	1	0.5	0	0	0
G1214	Mdx/utrn ·	M	21.9	0	1	0.5	0.5	0.5	0.5
G1215	Mdx/utrn ·	M	17.9	1	1	1	0.5	0.5	0.5
G1220	Mdx/utrn ·	M	12.9	1	1	1	1	1	1
G1352	Mdx/utrn ·	M	18	0.5	0.5	0.5	0	0	0

Mouse	Genotype	Sex	Weight	Clasp 1	Clasp 2	Clasp Avg	Gait 1	Gait 2	Gait Avg
G998	WT	M	22.3	0	0	0	0	0	0
G1037	WT	M	23.2	0	0	0	0	0	0
G1167	WT	M	26.6	0	0	0	0	0	0
G1168	WT	M	24.3	0	0	0	0	0	0
G1169	WT	M	23.7	1	0	-0.5	0.5	0.5	0.5
G1368	WT	M	18.2	0	0	0	0	0	0

Kyph 1	Kyph2	Kyph Avg	Ledge 1	Ledge 2	Ledge Avg	Wire 1	Wire 2	Wire Avg	Grip 1
2	0.5	1.25	2	1	1.5	9	120	64.5	0.5
2	0	1	0	1	0.5	34	5	19.5	0.3
2	2	2	2	2	2	120	62	91	1.3
2	2	2	1	1	1	120	95	107.5	1.3
2	2	2	1	1	1	120	120	120	1.5
3	3	3	2	2	2	120	120	120	1.2
1	1	1	0.5	0.5	0.5	120	5	62.5	1.1

Kyph 1	Kyph2	Kyph Avg	Ledge 1	Ledge 2	Ledge Avg	Wire 1	Wire 2	Wire Avg	Grip 1
0	0	0	0	0	0	120	120	120	1.2
0	0	0	0	0	0	120	120	120	2.2
0	0	0	0	0.5	0.25	120	120	120	1.8
0	0	0	0	0	0	120	109	114.5	1.9
0	0	0	1	0.5	0.75	120	120	120	1.7
0	0	0	0	0	0	120	120	120	1.5

Grip 2	Grip 3	Grip Avg
1.1	0.9	0.833333
0.9	0.9	0.7
1.4	0.9	1.2
1.2	1.2	1.233333
1.6	1.6	1.566667
1	0.7	0.966667
1	0.9	1

Grip 2	Grip 3	Grip Avg
1.2	1.7	1.366667
1.5	1.9	1.866667
2	2.2	2
2.3	2.2	2.133333
2.1	2	1.933333
1.8	1.6	1.633333

Mouse	Genotype	Sex	Weight	Clasp 1	Clasp 2	Clasp Avg	Gait 1	Gait 2	Gait Avg
G809	Mdx/utrn -	M	18.3	0	0	0	1	1	1
G810	Mdx/utrn -	M	18.5	0	0	0	1	0.5	0.75
G1213	Mdx/utrn -	M	16	1	1	1	1	1	1
G1214	Mdx/utrn -	M	21.5	0	0	0	0.5	0.5	0.5
G1215	Mdx/utrn -	M	16.7	1	1	1	1	1	1
G1352	Mdx/utrn -	M	20.2	0.5	0.5	0.5	0	0	0

Mouse	Genotype	Sex	Weight	Clasp 1	Clasp 2	Clasp Avg	Gait 1	Gait 2	Gait Avg
G998	WT	M	22.7	0	0	0	0	0	0
G1167	WT	M	28.1	0	0	0	0	0	0
G1168	WT	M	26.4	0	0	0	0	0	0
G1169	WT	M	25.2	0	0	0	0.5	0.5	0.5
G1368	WT	M	20.8	0	0	0	0	0	0

Kyph 1	Kyph2	Kyph Avg	Ledge 1	Ledge 2	Ledge Avg	Wire 1	Wire 2	Wire Avg	Grip 1
3	3	3	1	1	1	120	18	69	1.5
3	3	3	1	1	1	120	12	66	1.5
2	2	2	1	1	1	47	71	59	1.8
2	2	2	0.5	0.5	0.5	120	67	93.5	1.7
2	2	2	0.5	0.5	0.5	120	120	120	1.7
1	1	1	0.5	0.5	0.5	120	45	82.5	1

Kyph 1	Kyph2	Kyph Avg	Ledge 1	Ledge 2	Ledge Avg	Wire 1	Wire 2	Wire Avg	Grip 1
0	0	0	0.5	0	0.25	120	120	120	1.8
0	0	0	0.5	0	0.25	120	120	120	2.7
0	0	0	0	0	0	120	120	120	2.4
0	0	0	0	0	0	120	120	120	2
0	0	0	0	0	0	120	120	120	1.6

Grip 2	Grip 3	Grip Avg
1.2	1.1	1.266667
0.7	0.7	0.966667
1.3	1.1	1.4
1.2	1.1	1.333333
1.4	1.3	1.466667
0.8	0.7	0.833333

Grip 2	Grip 3	Grip Avg
1.6	1.1	1.5
2.2	2.6	2.5
2.1	2	2.166667
2	1.4	1.8
1.6	1.8	1.666667

Mouse	Genotype	Sex	Weight	Clasp 1	Clasp 2	Clasp Avg	Gait 1	Gait 2	Gait Avg
G810	Mdx/utrn -	M	16.7	2	1.5	1.75	2	2	2
G1215	Mdx/utrn -	M	14.3	1	1	1	1	1	1
G1214	Mdx/utrn -	M	21	1	1	1	1	1	1
G1352	Mdx/utrn -	M	20.8	0.25	0.5	0.375	0.5	0.5	0.5

Mouse	Genotype	Sex	Weight	Clasp 1	Clasp 2	Clasp Avg	Gait 1	Gait 2	Gait Avg
G998	WT	M	22.9	0	0	0	0	0	0
G1169	WT	M	24.9	0	0	0	0.5	0.5	0.5
G1168	WT	M	25.9	0	0	0	0.5	0.5	0.5
G1368	WT	M	21.2	0	0	0	0	0	0

Kyph 1	Kyph2	Kyph Avg	Ledge 1	Ledge 2	Ledge Avg	Wire 1	Wire 2	Wire Avg	Grip 1
3	3	3	2	2	2	21	120	70.5	1.1
2	2	2	2	2	2	120	120	120	1.5
2	2	2	1	1.2	1.1	120	120	120	1.6
2	2	2	0	0.25	0.125	120	38	79	1.2

Kyph 1	Kyph2	Kyph Avg	Ledge 1	Ledge 2	Ledge Avg	Wire 1	Wire 2	Wire Avg	Grip 1
0	0	0	0	0	0	120	120	120	2.5
0	0	0	0	0	0	120	120	120	2.5
0	0	0	0.5	0	0.25	120	92	106	1.6
0	0	0	0	0	0	120	120	120	1.4

Grip 2	Grip 3	Grip Avg
1.1	0.7	0.966667
1.5	1.4	1.466667
1.4	1.6	1.533333
0.9	1.1	1.066667

Grip 2	Grip 3	Grip Avg
1.9	1.7	2.033333
2	2.4	2.3
2.2	2.6	2.133333
1.8	2.1	1.766667

Mouse	Genotype	Sex	Weight	Clasp 1	Clasp 2	Clasp Avg	Gait 1	Gait 2	Gait Avg
G810	Mdx/utrn -	M	18.1	2	2	2	1.5	1.5	1.5
G1215	Mdx/utrn -	M	15.4	1	1	1	1	1	1
G1214	Mdx/utrn -	M	20.7	1	1	1	1	1	1
G1352	Mdx/utrn -	M							

Mouse	Genotype	Sex	Weight	Clasp 1	Clasp 2	Clasp Avg	Gait 1	Gait 2	Gait Avg
G998	WT	M	22.7	0	0	0	0	0	0
G1169	WT	M	24.8	0	0	0	0.5	0.5	0.5
G1168	WT	M	26.4	0	0	0	0	0	0

Kyph 1	Kyph2	Kyph Avg	Ledge 1	Ledge 2	Ledge Avg	Wire 1	Wire 2	Wire Avg	Grip 1
3	3	3	1	1	1	116	64	90	1.4
2	2	2	1	1.5	1.25	120	120	120	1.7
2	2	2	1	1	1	120	120	120	1.5

Kyph 1	Kyph2	Kyph Avg	Ledge 1	Ledge 2	Ledge Avg	Wire 1	Wire 2	Wire Avg	Grip 1
0	0	0	0	0	0	120	120	120	2.1
0	0	0	0	0	0	120	120	120	1.9
0	0	0	0	0	0	120	120	120	2.4

Grip 2	Grip 3	Grip Avg
0.8	1.2	1.133333
1.6	1.5	1.6
1.7	1.5	1.566667

Grip 2	Grip 3	Grip Avg
1.7	1.9	1.9
2.1	2.2	2.066667
2.1	2.3	2.266667

Mouse	Genotype	Sex	Weight	Clasp 1	Clasp 2	Clasp Avg	Gait 1	Gait 2	Gait Avg
G665	Mdx/utrn -	M	14.5	2.5	3	2.75	2	2	2
G1352	Mdx/utrn -	M							

Mouse	Genotype	Sex	Weight	Clasp 1	Clasp 2	Clasp Avg	Gait 1	Gait 2	Gait Avg
G998	WT	M	24.1	0	0	0	0	0	0

Kyph 1	Kyph2	Kyph Avg	Ledge 1	Ledge 2	Ledge Avg	Wire 1	Wire 2	Wire Avg	Grip 1
3	3	3	2	2	2	120	120	120	1

Kyph 1	Kyph2	Kyph Avg	Ledge 1	Ledge 2	Ledge Avg	Wire 1	Wire 2	Wire Avg	Grip 1
0	0	0	0	0	0	120	120	120	2.1

Grip 2	Grip 3	Grip Avg
1.2	1.2	1.133333

Grip 2	Grip 3	Grip Avg
1.7	1.9	1.9

Metabolism
WT 6 weeks Baseline

**G1035 6wks**	Time	MV	Time	VCO2	MVb/VCO2
	0:50:22	68.855	0:50:22	0.64499	106.753593
	0:50:24	36.787	0:50:24	0.65259	56.3707688
	0:50:26	41.534	0:50:26	0.64612	64.2821767
	0:50:28	16.564	0:50:28	0.65031	25.4709292
	0:50:30	21.686	0:50:30	0.65925	32.8949564
	0:50:32	25.799	0:50:32	0.65795	39.2111863
	0:50:34	25.469	0:50:34	0.68747	37.0474348
	0:50:36	25.746	0:50:36	0.73905	34.8366146
	0:50:38	23.307	0:50:38	0.79742	29.2280103
	0:50:40	21.387	0:50:40	0.82037	26.069944
	0:50:42	23.89	0:50:42	0.80992	29.4967404
	0:50:44	27.732	0:50:44	0.77523	35.7726094
	0:50:46	23.478	0:50:46	0.80389	29.2054883
	0:50:48	19.637	0:50:48	0.80728	24.3248935
	0:50:50	21.784	0:50:50	0.80735	26.9821019
	0:50:52	26.727	0:50:52	0.78585	34.0103073
					39.4973597

Challenge	Time	MV	Time	VCO2	MVb/VCO2
	1:35:22	186.69	1:35:22	0.64592	289.029601
	1:35:24	178.21	1:35:24	0.77805	229.046976
	1:35:26	182.11	1:35:26	0.81187	224.309311
	1:35:28	193.65	1:35:28	0.84722	228.571091
	1:35:30	192.16	1:35:30	0.88582	216.928947
	1:35:32	182.93	1:35:32	0.94395	193.792044
	1:35:34	195.59	1:35:34	1.0033	194.946676
	1:35:36	187.66	1:35:36	1.0002	187.622476
	1:35:38	180.99	1:35:38	1.0276	176.128844
	1:35:40	189.53	1:35:40	1.0781	175.800019
	1:35:42	205.55	1:35:42	1.0686	192.354483
	1:35:44	182.91	1:35:44	1.0813	169.157496
	1:35:46	191.45	1:35:46	1.0675	179.344262
	1:35:48	214.91	1:35:48	1.1038	194.700127
	1:35:50	181.3	1:35:50	1.1151	162.586315
	1:35:52	183.22	1:35:52	1.118	163.881932
					198.637537

Metabolism

mdx/utrn 6 w	Baseline				
**G1023 6wks**	Time	MV	Time	VCO2	MVb/VCO2
	1:14:26	34.088	1:14:26	0.60176	56.6471683
	1:14:28	31.732	1:14:28	0.59585	53.2550138
	1:14:30	33.564	1:14:30	0.59222	56.6748843
	1:14:32	32.469	1:14:32	0.5724	56.7243187
	1:14:34	31.266	1:14:34	0.56696	55.1467476
	1:14:36	33.054	1:14:36	0.56175	58.8411215
	1:14:38	33.3	1:14:38	0.5592	59.5493562
	1:14:40	36.016	1:14:40	0.55004	65.4788743
	1:14:42	36.653	1:14:42	0.56991	64.3136636
	1:14:44	36.122	1:14:44	0.55568	65.0050389
	1:14:46	33.07	1:14:46	0.55205	59.9039942
	1:14:48	34.375	1:14:48	0.55652	61.7677711
	1:14:50	37.364	1:14:50	0.55221	67.6626646
	1:14:52	37.855	1:14:52	0.55172	68.6127021
	1:14:54	35.068	1:14:54	0.55217	63.5094264
	1:14:56	33.708	1:14:56	0.55079	61.1993682
					60.8932571

Challenge	Time	MV	Time	VCO2	MVb/VCO2
	1:21:56	50.811	1:21:56	0.91112	55.7676267
	1:21:58	51.261	1:21:58	0.93153	55.0288235
	1:22:00	46.979	1:22:00	0.89907	52.2528835
	1:22:02	49.488	1:22:02	0.95541	51.7976576
	1:22:04	46.891	1:22:04	0.95825	48.9339943
	1:22:06	48.414	1:22:06	0.94647	51.152176
	1:22:08	49.096	1:22:08	0.92091	53.3124844
	1:22:10	49.037	1:22:10	0.92342	53.1036798
	1:22:12	47.337	1:22:12	0.91495	51.7372534
	1:22:14	55.274	1:22:14	0.91492	60.4140253
	1:22:16	46.973	1:22:16	0.93314	50.3386416

1:22:18	50.783	1:22:18	0.89449	56.7731333
1:22:20	60.792	1:22:20	0.92715	65.5686782
1:22:22	66.823	1:22:22	0.96065	69.5601936
1:22:24	59.891	1:22:24	0.94016	63.7029867
1:22:26	49.297	1:22:26	0.87924	56.0677403
				55.9694986

G1036 6wks	Time	MV	Time	VCO2	MVb/VCO2
	0:47:22	30.976	0:47:22	0.67515	45.8801748
	0:47:24	30.564	0:47:24	0.66723	45.8072928
	0:47:26	28.785	0:47:26	0.66628	43.2025575
	0:47:28	31.689	0:47:28	0.67013	47.2878397
	0:47:30	33.234	0:47:30	0.67238	49.4274071
	0:47:32	32.298	0:47:32	0.66997	48.2081287
	0:47:34	34.923	0:47:34	0.667	52.3583208
	0:47:36	40.832	0:47:36	0.6584	62.0170109
	0:47:38	65.085	0:47:38	0.67078	97.0288321
	0:47:40	38.288	0:47:40	0.66028	57.9875204
	0:47:42	10.86	0:47:42	0.65678	16.5352173
	0:47:44	18.126	0:47:44	0.65333	27.7440191
	0:47:46	28.218	0:47:46	0.66354	42.526449
	0:47:48	28.149	0:47:48	0.66738	42.1783691
	0:47:50	27.952	0:47:50	0.66936	41.7592925
	0:47:52	34.343	0:47:52	0.69169	49.6508552
					48.0999554

	Time	MV	Time	VCO2	MVb/VCO2
	1:43:26	169.49	1:43:26	1.3695	123.760497
	1:43:28	173.47	1:43:28	1.4122	122.836709
	1:43:30	179.26	1:43:30	1.4186	126.364021
	1:43:32	168.47	1:43:32	1.4581	115.540772
	1:43:34	185.68	1:43:34	1.4888	124.717894
	1:43:36	174.27	1:43:36	1.5197	114.673949
	1:43:38	176.37	1:43:38	1.4782	119.314031
	1:43:40	167.74	1:43:40	1.5229	110.145118
	1:43:42	171.21	1:43:42	1.492	114.752011
	1:43:44	175.69	1:43:44	1.4941	117.589184
	1:43:46	175.6	1:43:46	1.497	117.301269
	1:43:48	151.99	1:43:48	1.5085	100.755718
	1:43:50	151.42	1:43:50	1.5111	100.205149
	1:43:52	146.91	1:43:52	1.5327	95.85046
	1:43:54	171.61	1:43:54	1.582	108.476612
					114.152226

G1220 6wks	Time	MV	Time	VCO2	MVb/VCO2
	1:05:56	17.459	1:05:56	0.4683	37.2816571
	1:05:58	19.501	1:05:58	0.47004	41.4879585
	1:06:00	18.051	1:06:00	0.47013	38.3957629
	1:06:02	20.865	1:06:02	0.47886	43.5722341
	1:06:04	16.546	1:06:04	0.47416	34.895394
	1:06:06	29.032	1:06:06	0.4609	62.9898026
	1:06:08	23.757	1:06:08	0.45058	52.7253762
	1:06:10	23.704	1:06:10	0.44725	52.999441
	1:06:12	28.552	1:06:12	0.44779	63.7620313
	1:06:14	24.947	1:06:14	0.44792	55.6952134
	1:06:16	19.142	1:06:16	0.44712	42.8117731
	1:06:18	19.229	1:06:18	0.44081	43.6219686
	1:06:20	20.39	1:06:20	0.41528	49.0994028
	1:06:22	26.098	1:06:22	0.45645	57.1760324
	1:06:24	17.651	1:06:24	0.4983	35.4224363
	1:06:26	19.716	1:06:26	0.49771	39.6134295
					46.9718696

Time	MV	Time	VCO2	MVb/VCO2
1:39:42	60.796	1:39:42	0.5326	114.149456
1:39:44	62.209	1:39:44	0.52367	118.794279
1:39:46	61.232	1:39:46	0.5984	102.326203
1:39:48	65.062	1:39:48	0.60763	107.075029
1:39:50	63.752	1:39:50	0.65298	97.632393
1:39:52	60.631	1:39:52	0.65533	92.5197992
1:39:54	60.633	1:39:54	0.62679	96.7357488
1:39:56	61.84	1:39:56	0.63304	97.6873499
1:39:58	61.714	1:39:58	0.64832	95.1906466
1:40:00	65.105	1:40:00	0.66117	98.46938
1:40:02	61.279	1:40:02	0.68044	90.0579037

1:40:04	62.138	1:40:04	0.67832	91.6057318
1:40:06	63.698	1:40:06	0.70928	89.8065644
1:40:08	63.247	1:40:08	0.71839	88.0399226
1:40:10	63.665	1:40:10	0.68106	93.4792823
				98.2379793

G1037 6wks	Time	MV	Time	VCO2
	0:36:52	36.454	0:36:52	0.70889
	0:36:54	27.66	0:36:54	0.71458
	0:36:56	31.472	0:36:56	0.71324
	0:36:58	26.114	0:36:58	0.70855
	0:37:00	28.86	0:37:00	0.70569
	0:37:02	27.678	0:37:02	0.70916
	0:37:04	24.965	0:37:04	0.71604
	0:37:06	31.002	0:37:06	0.72533
	0:37:08	24.121	0:37:08	0.7294
	0:37:10	27.656	0:37:10	0.74564
	0:37:12	35.117	0:37:12	0.75365
	0:37:14	24.9	0:37:14	0.73615
	0:37:16	33.272	0:37:16	0.75126
	0:37:18	25.637	0:37:18	0.73423
	0:37:20	25.456	0:37:20	0.72717
	0:37:22	28.642	0:37:22	0.74211

Time	MV	Time	VCO2
1:36:52	174.15	1:36:52	1.1887
1:36:54	171.26	1:36:54	1.261
1:36:56	169.97	1:36:56	1.2999
1:36:58	171.57	1:36:58	1.2323
1:37:00	166.81	1:37:00	1.2408
1:37:02	171.95	1:37:02	1.221
1:37:04	190.06	1:37:04	1.2477
1:37:06	168.42	1:37:06	1.2542
1:37:08	165.6	1:37:08	1.2668
1:37:10	182.4	1:37:10	1.2515
1:37:12	178.3	1:37:12	1.2544
1:37:14	173.87	1:37:14	1.2418
1:37:16	166.88	1:37:16	1.2523
1:37:18	192.9	1:37:18	1.2632
1:37:20	200.67	1:37:20	1.2722
1:37:22	177.61	1:37:22	1.2752

G1222 6wks	Time	MV	Time	VCO2
	1:21:42	28.16	1:21:42	0.55233
	1:21:44	29.96	1:21:44	0.56233
	1:21:46	25.195	1:21:46	0.57919
	1:21:48	25.544	1:21:48	0.58608
	1:21:50	22.782	1:21:50	0.59738
	1:21:52	25.156	1:21:52	0.60021
	1:21:54	22.746	1:21:54	0.58969
	1:21:56	26.152	1:21:56	0.57158
	1:21:58	22.703	1:21:58	0.57457
	1:22:00	23.392	1:22:00	0.57231
	1:22:02	23.85	1:22:02	0.588
	1:22:04	22.083	1:22:04	0.56306
	1:22:06	26.456	1:22:06	0.56246
	1:22:08	23.577	1:22:08	0.56918
	1:22:10	24.538	1:22:10	0.56198

Time	MV	Time	VCO2
1:40:26	45.597	1:40:26	0.8493
1:40:28	46.068	1:40:28	0.84104
1:40:30	44.198	1:40:30	0.86478
1:40:32	46.261	1:40:32	0.90241
1:40:34	51.539	1:40:34	0.90704
1:40:36	47.368	1:40:36	0.88334
1:40:38	48.948	1:40:38	0.91709
1:40:40	41.371	1:40:40	0.89774
1:40:42	43.968	1:40:42	0.8734
1:40:44	47.046	1:40:44	0.9469
1:40:46	45.103	1:40:46	0.91358

1:40:48	43.494	1:40:48	0.90813
1:40:50	44.666	1:40:50	0.93818
1:40:52	44.035	1:40:52	0.9466
1:40:54	42.398	1:40:54	0.90985
1:40:56	42.643	1:40:56	0.90184

MVb/VCO2	G1167	Time	MV	Time
51.4240573		1:27:30	42.237	1:27:30
38.7080523		1:27:32	41.913	1:27:32
44.1253996		1:27:34	40.406	1:27:34
36.8555501		1:27:36	43.885	1:27:36
40.8961442		1:27:38	45.387	1:27:38
39.0292741		1:27:40	43.156	1:27:40
34.8653706		1:27:42	42.881	1:27:42
42.7419244		1:27:44	42.491	1:27:44
33.0696463		1:27:46	43.682	1:27:46
37.0902849		1:27:48	47.15	1:27:48
46.5959		1:27:50	40.731	1:27:50
33.8246281		1:27:52	39.862	1:27:52
44.2882624		1:27:54	38.163	1:27:54
34.9168517		1:27:56	57.606	1:27:56
35.0069447		1:27:58	49.961	1:27:58
38.5953565		1:28:00	46.277	1:28:00
39.5021029				

MVb/VCO2	Time	MV	Time
146.504585	1:38:44	188.99	1:38:44
135.812847	1:38:46	194.84	1:38:46
130.756212	1:38:48	195.84	1:38:48
139.227461	1:38:50	174.81	1:38:50
134.43746	1:38:52	192.97	1:38:52
140.827191	1:38:54	188.61	1:38:54
152.328284	1:38:56	196.78	1:38:56
134.284803	1:38:58	179.22	1:38:58
130.723082	1:39:00	186.12	1:39:00
145.745106	1:39:02	195.43	1:39:02
142.139668	1:39:04	179.56	1:39:04
140.014495	1:39:06	196.23	1:39:06
133.258804	1:39:08	185.68	1:39:08
152.70741	1:39:10	177.04	1:39:10
157.734633	1:39:12	182.55	1:39:12
139.280113	1:39:14	203.02	1:39:14
140.986385			

MVb/VCO2		Time	MV	Time
50.9840132	**G1213 7wks**	1:42:38	37.059	1:42:38
53.2783241		1:42:40	29.342	1:42:40
43.5004057		1:42:42	27.709	1:42:42
43.5844936		1:42:44	29.515	1:42:44
38.1365295		1:42:46	42.997	1:42:46
41.9119975		1:42:48	50.919	1:42:48
38.5728094		1:42:50	49.857	1:42:50
45.7538752		1:42:52	37.813	1:42:52
39.5130271		1:42:54	38.925	1:42:54
40.8729535		1:42:56	46.676	1:42:56
40.5612245		1:42:58	38.991	1:42:58
39.2196214		1:43:00	39.372	1:43:00
47.0362337		1:43:02	30.288	1:43:02
41.4227485		1:43:04	34.811	1:43:04
43.6634756		1:43:06	35.614	1:43:06
43.2007822		1:43:08	37.462	1:43:08

MVb/VCO2	Time	MV	Time
53.6877428	1:57:38	73.635	1:57:38
54.7750404	1:57:40	80.363	1:57:40
51.1089526	1:57:42	73.904	1:57:42
51.2638379	1:57:44	70.927	1:57:44
56.8210884	1:57:46	72.736	1:57:46
53.6237462	1:57:48	71.102	1:57:48
53.3731695	1:57:50	71.357	1:57:50
46.0834986	1:57:52	69.717	1:57:52
50.3411953	1:57:54	68.625	1:57:54
49.6842328	1:57:56	70.758	1:57:56
49.3695133	1:57:58	68.476	1:57:58

47.894024	1:58:00	67.743	1:58:00
47.6092008	1:58:02	72.519	1:58:02
46.5191211	1:58:04	70.707	1:58:04
46.5988899	1:58:06	70.686	1:58:06
47.2844407	1:58:08	71.49	1:58:08
50.3773559			

VCO2	MVb/VCO2	G1168	Time	MV
0.91576	46.1223465		1:28:14	37.905
0.90935	46.091164		1:28:16	39.222
0.90841	44.4799155		1:28:18	42.599
0.88776	49.4334054		1:28:20	41.641
0.86055	52.7418511		1:28:22	39.155
0.88796	48.6012883		1:28:24	45.226
0.85337	50.2490127		1:28:26	43.238
0.86257	49.2609295		1:28:28	40.099
0.86849	50.2964916		1:28:30	39.693
0.87238	54.0475481		1:28:32	44.173
0.86298	47.1980811		1:28:34	39.279
0.86575	46.043315		1:28:36	38.125
0.87308	43.7107711		1:28:38	39.182
0.86451	66.6342784		1:28:40	39.369
0.8638	57.8386201		1:28:42	39.388
0.86041	53.7848235		1:28:44	37.223
	50.4083651			

VCO2	MVb/VCO2		Time	MV
0.97544	193.748462		1:39:30	111.78
1.1317	172.165768		1:39:32	103.99
1.2811	152.868629		1:39:34	101.12
1.3574	128.782967		1:39:36	108.06
1.4188	136.009304		1:39:38	99.032
1.491	126.498994		1:39:40	95.54
1.5458	127.29978		1:39:42	98.573
1.5184	118.032139		1:39:44	98.067
1.5732	118.306636		1:39:46	97.072
1.6255	120.227622		1:39:48	99.444
1.6001	112.217986		1:39:50	99.672
1.665	117.855856		1:39:52	99.115
1.6419	113.088495		1:39:54	98.89
1.6792	105.431158		1:39:56	97.16
1.6869	108.216255		1:39:58	100.04
1.7632	115.142922			
	129.118311			

VCO2	MVb/VCO2			Time	MV
0.73993	50.0844674		**G1214 7wks**	1:31:30	28.851
0.74065	39.616553			1:31:32	25.742
0.74987	36.9517383			1:31:34	25.196
0.74083	39.8404492			1:31:36	25.833
0.73929	58.1598561			1:31:38	23.946
0.74091	68.7249463			1:31:40	22.424
0.76468	65.1998221			1:31:42	22.272
0.7558	50.0304313			1:31:44	22.449
0.76937	50.5933426			1:31:46	22.891
0.79903	58.4158292			1:31:48	23.951
0.82834	47.0712509			1:31:50	22.175
0.90785	43.3683979			1:31:52	21.663
0.98636	30.7068413			1:31:54	22.054
0.98642	35.2902415			1:31:56	23.149
0.97855	36.3946656			1:31:58	27.566
0.985	38.0324873			1:32:00	16.004
	46.7800825				

VCO2	MVb/VCO2	Time	MV
0.26244	280.578418	1:49:30	94.444
0.40845	196.751132	1:49:32	92.919
0.53309	138.633251	1:49:34	85.426
0.53099	133.57502	1:49:36	84.105
0.58731	123.84601	1:49:38	90.545
0.61284	116.020495	1:49:40	87.544
0.69361	102.877698	1:49:42	85.833
0.57068	122.164786	1:49:44	85.587
0.6212	110.471668	1:49:46	86.759
0.66282	106.752965	1:49:48	84.194
0.66834	102.456833	1:49:50	82.763

0.6438	105.223672	1:49:52	91.302
0.67756	107.029636	1:49:54	85.552
0.70619	100.124612	1:49:56	83.94
0.66985	105.525118	1:49:58	109.46
0.65334	109.422353	1:50:00	98.7
	128.840854		

Time	VCO2	MVb/VCO2
1:28:14	0.80929	46.8373513
1:28:16	0.80583	48.672797
1:28:18	0.79853	53.3467747
1:28:20	0.80991	51.4143547
1:28:22	0.81773	47.8825529
1:28:24	0.82492	54.8247103
1:28:26	0.81705	52.91965
1:28:28	0.81836	48.9992179
1:28:30	0.82632	48.0358699
1:28:32	0.81559	54.1607916
1:28:34	0.79067	49.6781211
1:28:36	0.8083	47.1668935
1:28:38	0.80409	48.7283762
1:28:40	0.81578	48.2593346
1:28:42	0.82436	47.7800961
1:28:44	0.82028	45.3784074
		49.6303312

G1169

Time
1:13:24
1:13:26
1:13:28
1:13:30
1:13:32
1:13:34
1:13:36
1:13:38
1:13:40
1:13:42
1:13:44
1:13:46
1:13:48
1:13:50
1:13:52
1:13:54

Time	VCO2	MVb/VCO2
1:39:30	1.2532	89.1956591
1:39:32	1.2674	82.0498659
1:39:34	1.3121	77.0672967
1:39:36	1.3704	78.8528897
1:39:38	1.3615	72.737422
1:39:40	1.3801	69.2268676
1:39:42	1.3868	71.0794635
1:39:44	1.3475	72.7769944
1:39:46	1.4288	67.9395297
1:39:48	1.3965	71.2094522
1:39:50	1.4227	70.0583398
1:39:52	1.3979	70.9027827
1:39:54	1.4235	69.4696171
1:39:56	1.4189	68.4755797
1:39:58	1.4147	70.7146391
		73.4504266

Time
1:40:14
1:40:16
1:40:18
1:40:20
1:40:22
1:40:24
1:40:26
1:40:28
1:40:30
1:40:32
1:40:34
1:40:36
1:40:38
1:40:40
1:40:42
1:40:44

Time	VCO2	MVb/VCO2
1:31:30	0.66814	43.1810698
1:31:32	0.68425	37.6207526
1:31:34	0.67709	37.2121874
1:31:36	0.6811	37.9283512
1:31:38	0.67887	35.2733218
1:31:40	0.68666	32.6566277
1:31:42	0.71637	31.0900791
1:31:44	0.74082	30.3029076
1:31:46	0.74789	30.6074423
1:31:48	0.7682	31.1780786
1:31:50	0.78314	28.3154991
1:31:52	0.80637	26.8648387
1:31:54	0.81927	26.9190865
1:31:56	0.82132	28.1851166
1:31:58	0.8181	33.6951473
1:32:00	0.85096	18.8069945
		31.8648438

**G1215 7wks**	Time
	1:08:14
	1:08:16
	1:08:18
	1:08:20
	1:08:22
	1:08:24
	1:08:26
	1:08:28
	1:08:30
	1:08:32
	1:08:34
	1:08:36
	1:08:38
	1:08:40
	1:08:42
	1:08:44

Time	VCO2	MVb/VCO2
1:49:30	0.47848	197.383381
1:49:32	0.56755	163.719496
1:49:34	0.65706	130.01248
1:49:36	0.67366	124.847846
1:49:38	0.74714	121.1888
1:49:40	0.73322	119.396634
1:49:42	0.76046	112.869842
1:49:44	0.79235	108.016659
1:49:46	0.77569	111.847516
1:49:48	0.78693	106.990457
1:49:50	0.79366	104.28017

Time
1:50:14
1:50:16
1:50:18
1:50:20
1:50:22
1:50:24
1:50:26
1:50:28
1:50:30
1:50:32
1:50:34

1:49:52	0.81642	111.832145	1:50:36
1:49:54	0.76133	112.37177	1:50:38
1:49:56	0.82163	102.162774	1:50:40
1:49:58	0.8316	131.625782	1:50:42
1:50:00	0.81549	121.031527	1:50:44
		123.72358	

MV	Time	VCO2	MVb/VCO2
23.803	1:13:24	0.88931	26.7656948
25.189	1:13:26	0.91859	27.4213741
24.418	1:13:28	0.91834	26.5892807
24.692	1:13:30	0.92453	26.7076244
23.3	1:13:32	0.9253	25.1810224
24.93	1:13:34	0.92666	26.9030712
35.493	1:13:36	0.92724	38.2781157
27.938	1:13:38	0.92597	30.1716038
20.076	1:13:40	0.92606	21.6789409
25.701	1:13:42	0.92899	27.6655292
25.915	1:13:44	0.91424	28.3459485
45.527	1:13:46	0.92799	49.0597959
18.878	1:13:48	0.9029	20.9081847
25.347	1:13:50	0.90888	27.8881701
26.327	1:13:52	0.9296	28.3207831
23.581	1:13:54	0.92323	25.5418476
			28.5891867

MV	Time	VCO2	MVb/VCO2
165.61	1:40:14	1.986	83.388721
175.04	1:40:16	1.9888	88.0128721
168.98	1:40:18	2.0274	83.3481306
177.11	1:40:20	2.1079	84.0220124
181.26	1:40:22	2.071	87.5229358
178.44	1:40:24	2.0395	87.4920324
188.72	1:40:26	2.1218	88.94335
216.42	1:40:28	2.1129	102.427943
185.43	1:40:30	2.0311	91.2953572
162.48	1:40:32	2.1141	76.8553995
166.59	1:40:34	2.1365	77.9733209
175.49	1:40:36	2.1172	82.8877763
161.72	1:40:38	2.1299	75.9284473
169.88	1:40:40	2.1953	77.3835011
190.95	1:40:42	2.1238	89.909596
170.91	1:40:44	2.0906	81.7516502
			84.9464403

MV	Time	VCO2	MVb/VCO2
30.41	1:08:14	0.71172	42.7274771
28.123	1:08:16	0.70149	40.0903791
26.744	1:08:18	0.72027	37.1305205
26.202	1:08:20	0.71073	36.8663205
27.422	1:08:22	0.71714	38.2380009
30.721	1:08:24	0.70628	43.4969134
30.331	1:08:26	0.70637	42.9392528
27.798	1:08:28	0.72854	38.1557636
29.11	1:08:30	0.72179	40.33029
28.254	1:08:32	0.70955	39.819604
27.18	1:08:34	0.69699	38.9962553
38.137	1:08:36	0.68731	55.4873347
33.067	1:08:38	0.69191	47.7908977
30.152	1:08:40	0.68508	44.0123781
30.465	1:08:42	0.68077	44.7507969
26.273	1:08:44	0.67088	39.1619962
			41.8746363

MV	Time	VCO2	MVb/VCO2
49.733	1:50:14	0.54729	90.8713845
57.893	1:50:16	0.5947	97.3482428
55.333	1:50:18	0.62533	88.4860794
53.866	1:50:20	0.56537	95.2756602
54.077	1:50:22	0.60363	89.586336
56.34	1:50:24	0.58717	95.9517687
54.42	1:50:26	0.62061	87.6879199
56.248	1:50:28	0.62507	89.9867215
54.598	1:50:30	0.58969	92.5876308
54.013	1:50:32	0.65696	82.2165733
54.493	1:50:34	0.66182	82.338098

53.225	1:50:36	0.75656	70.3513271
54.782	1:50:38	0.71746	76.3554763
54.359	1:50:40	0.6883	78.9757373
56.449	1:50:42	0.72473	77.8896969
51.771	1:50:44	0.70035	73.9216106
			85.6143914

Metabolism

WT 6 weeks	End stage				
**G1035 7wks**	Time	MV	Time	VCO2	MVb/VCO2
	0:53:56	17.323	0:53:56	0.56728	30.5369482
	0:53:58	18.268	0:53:58	0.56871	32.1218196
	0:54:00	22.809	0:54:00	0.572	39.8758741
	0:54:02	22.87	0:54:02	0.56754	40.2967192
	0:54:04	23.497	0:54:04	0.57317	40.9948183
	0:54:06	13.899	0:54:06	0.56169	24.7449661
	0:54:08	18.424	0:54:08	0.57123	32.253208
	0:54:10	22.326	0:54:10	0.56191	39.7323415
	0:54:12	16.35	0:54:12	0.57266	28.5509727
	0:54:14	17.908	0:54:14	0.54761	32.7021055
	0:54:16	29.036	0:54:16	0.56424	51.4603715
	0:54:18	18.275	0:54:18	0.55422	32.9742701
	0:54:20	15.693	0:54:20	0.56988	27.5373763
	0:54:22	16.291	0:54:22	0.55638	29.280348
	0:54:24	16.511	0:54:24	0.56939	28.9976993
	0:54:26	17.619	0:54:26	0.54588	32.2763245
					34.0210102

Challenge	Time	MV	Time	VCO2	MVb/VCO2
	1:42:40	180.19	1:42:40	0.72077	249.996531
	1:42:42	161.6	1:42:42	0.81195	199.027034
	1:42:44	166.57	1:42:44	0.93601	177.9575
	1:42:46	160.53	1:42:46	0.93933	170.898406
	1:42:48	176.88	1:42:48	0.95668	184.889409
	1:42:50	164.11	1:42:50	0.99642	164.699625
	1:42:52	169.35	1:42:52	1.0248	165.251756
	1:42:54	168.6	1:42:54	1.0974	153.635867
	1:42:56	161.72	1:42:56	1.1358	142.384223
	1:42:58	188.68	1:42:58	1.1224	168.104063
	1:43:00	190.5	1:43:00	1.0828	175.932767
	1:43:02	182.59	1:43:02	1.1627	157.039649
	1:43:04	178.42	1:43:04	1.1787	151.370154
	1:43:06	163.43	1:43:06	1.1778	138.758703
	1:43:08	156	1:43:08	1.1191	139.39773
	1:43:10	165.78	1:43:10	1.0828	153.103066
					168.277905

Metabolism

mdx/utrn 6 w	Baseline				
**G1023 7wks**	Time	MV	Time	VCO2	MVb/VCO2
	0:05:08	27.888	0:05:08	0.52404	53.2173117
	0:05:10	25.976	0:05:10	0.51774	50.171901
	0:05:12	28.738	0:05:12	0.49264	58.3346866
	0:05:14	27.992	0:05:14	0.4901	57.1148745
	0:05:16	27.995	0:05:16	0.4708	59.4626168
	0:05:18	30.072	0:05:18	0.47476	63.3414778
	0:05:20	28.388	0:05:20	0.46509	61.0376486
	0:05:22	30.12	0:05:22	0.4661	64.6213259
	0:05:24	24.637	0:05:24	0.46789	52.6555387
	0:05:26	25.603	0:05:26	0.46684	54.8432011
	0:05:28	26.02	0:05:28	0.475	54.7789474
	0:05:30	26.104	0:05:30	0.47675	54.754064
	0:05:32	26.342	0:05:32	0.49927	52.7610311
	0:05:34	28.473	0:05:34	0.48727	58.4337226
	0:05:36	30.515	0:05:36	0.47491	64.2542798
					57.3188418

Challenge	Time	MV	Time	VCO2	MVb/VCO2
	1:38:08	31.964	1:38:08	0.54581	58.5625034
	1:38:10	29.427	1:38:10	0.5685	51.762533
	1:38:12	32.245	1:38:12	0.56026	57.5536358
	1:38:14	31.396	1:38:14	0.56074	55.9902985
	1:38:16	30.916	1:38:16	0.61339	50.401865
	1:38:18	26.818	1:38:18	0.63728	42.0819734
	1:38:20	25.384	1:38:20	0.66401	38.2283399
	1:38:22	29.327	1:38:22	0.72953	40.1998547
	1:38:24	29.161	1:38:24	0.74738	39.0176349
	1:38:26	29.802	1:38:26	0.75285	39.5855748
	1:38:28	28.209	1:38:28	0.73652	38.3003856

1:38:30	25.724	1:38:30	0.72849	35.3113975
1:38:32	28.416	1:38:32	0.69383	40.9552772
1:38:34	33.114	1:38:34	0.71663	46.2079455
1:38:36	30.596	1:38:36	0.71431	42.8329437
				45.1328109

G1037 8wks	Time	MV	Time	VCO2	MVb/VCO2
	0:55:22	24.171	0:55:22	0.88388	27.3464724
	0:55:24	32.263	0:55:24	0.87655	36.8067994
	0:55:26	32.276	0:55:26	0.86893	37.1445341
	0:55:28	29.611	0:55:28	0.87211	33.9532857
	0:55:30	24.174	0:55:30	0.87454	27.6419603
	0:55:32	25.242	0:55:32	0.89267	28.2769669
	0:55:34	27.888	0:55:34	0.94422	29.5354896
	0:55:36	32.589	0:55:36	0.96535	33.7587404
	0:55:38	22.326	0:55:38	0.96399	23.1599913
	0:55:40	25.799	0:55:40	0.94617	27.2667702
	0:55:42	29.548	0:55:42	0.93076	31.7461
	0:55:44	28.992	0:55:44	0.90885	31.8996534
	0:55:46	26.326	0:55:46	0.89871	29.2930979
	0:55:48	24.766	0:55:48	0.88568	27.9626953
	0:55:50	26.832	0:55:50	0.90382	29.6873271
	0:55:52	26.218	0:55:52	0.92811	28.2488067
					30.2330432

Time	MV	Time	VCO2	MVb/VCO2
1:47:52	166.61	1:47:52	1.7925	92.9483961
1:47:54	169.01	1:47:54	1.864	90.6706009
1:47:56	172.75	1:47:56	1.9065	90.6110674
1:47:58	208.45	1:47:58	1.8784	110.972104
1:48:00	197.75	1:48:00	1.8657	105.992389
1:48:02	186.79	1:48:02	1.8829	99.2033565
1:48:04	190.74	1:48:04	1.8636	102.35029
1:48:06	201.12	1:48:06	1.8996	105.874921
1:48:08	182.36	1:48:08	1.8967	96.1459377
1:48:10	181.08	1:48:10	1.978	91.5470172
1:48:12	188.72	1:48:12	2.0099	93.8952187
1:48:14	193.87	1:48:14	1.9774	98.0428846
1:48:16	182.37	1:48:16	1.9876	91.753874
1:48:18	168.64	1:48:18	1.9998	84.3284328
1:48:20	171.02	1:48:20	2.008	85.1693227
				95.9670541

G1220 8wks	Time	MV	Time	VCO2	MVb/VCO2
	0:42:34	22.683	0:42:34	0.35703	61.4732107
	0:42:36	23.114	0:42:36	0.36899	64.234104
	0:42:38	22.574	0:42:38	0.35984	62.5474495
	0:42:40	21.935	0:42:40	0.36091	59.6659685
	0:42:42	22.242	0:42:42	0.36763	60.2960312
	0:42:44	37.671	0:42:44	0.36888	101.25252
	0:42:46	23.797	0:42:46	0.37205	65.4843148
	0:42:48	23.24	0:42:48	0.3634	64.1492768
	0:42:50	20.162	0:42:50	0.36228	55.8953176
	0:42:52	23.065	0:42:52	0.36071	63.4909712
	0:42:54	22.609	0:42:54	0.36328	61.3541384
	0:42:56	22.847	0:42:56	0.3685	60.7471417
	0:42:58	25.578	0:42:58	0.3761	66.226503
	0:43:00	22.481	0:43:00	0.38622	58.3210107
	0:43:02	24.013	0:43:02	0.38547	61.4426079
	0:43:04	24.307	0:43:04	0.39082	62.1948723
					64.2984649

Time	MV	Time	VCO2	MVb/VCO2
1:53:38	26.932	1:53:48	4.1753	6.45031495
1:53:52	46.634	1:53:50	4.2801	10.8955398
1:53:58	10.246	1:53:52	4.491	2.28145179
1:54:00	14.971	1:53:54	4.6478	3.22109385
1:54:02	18.542	1:53:56	4.8089	3.85576743
1:54:06	25.02	1:53:58	4.9651	5.03917343
1:54:10	7.9073	1:54:00	5.0355	1.57031079
1:54:12	13.156	1:54:02	5.0353	2.61275396
1:54:14	19.112	1:54:04	5.0555	3.78043715
		1:54:06	5.1848	0
		1:54:08	5.1786	0

1:54:10	5.2471	0
1:54:12	5.2619	0
1:54:14	5.3368	0
1:54:16	5.3635	0
1:54:18	5.3366	0
		2.4816777

G1036 7wks	Time	MV	Time	VCO2
	1:17:10	38.286	1:17:10	0.81026
	1:17:12	28.048	1:17:12	0.83152
	1:17:14	40.521	1:17:14	0.83518
	1:17:16	40.984	1:17:16	0.83249
	1:17:18	38.417	1:17:18	0.83045
	1:17:20	39.841	1:17:20	0.85422
	1:17:22	39.562	1:17:22	0.84785
	1:17:24	40.077	1:17:24	0.85494
	1:17:26	39.411	1:17:26	0.85467
	1:17:28	38.097	1:17:28	0.83893
	1:17:30	46.795	1:17:30	0.8493
	1:17:32	43.874	1:17:32	0.85533
	1:17:34	39.758	1:17:34	0.84704
	1:17:36	39.241	1:17:36	0.8442
	1:17:38	39.213	1:17:38	0.84101
	1:17:40	38.966	1:17:40	0.83961

Time	MV	Time	VCO2
1:43:26	169.49	1:43:26	1.3695
1:43:28	173.47	1:43:28	1.4122
1:43:30	179.26	1:43:30	1.4186
1:43:32	168.47	1:43:32	1.4581
1:43:34	185.68	1:43:34	1.4888
1:43:36	174.27	1:43:36	1.5197
1:43:38	176.37	1:43:38	1.4782
1:43:40	167.74	1:43:40	1.5229
1:43:42	171.21	1:43:42	1.492
1:43:44	175.69	1:43:44	1.4941
1:43:46	175.6	1:43:46	1.497
1:43:48	151.99	1:43:48	1.5085
1:43:50	151.42	1:43:50	1.5111
1:43:52	146.91	1:43:52	1.5327
1:43:54	171.61	1:43:54	1.582
1:44:40	143.24	1:44:40	1.3763

G1222 7wks	Time	MV	Time	VCO2
	0:17:54	30.171	0:17:54	0.68677
	0:17:56	30.134	0:17:56	0.70037
	0:17:58	28.348	0:17:58	0.70742
	0:18:00	30.25	0:18:00	0.72275
	0:18:02	30.218	0:18:02	0.71344
	0:18:04	28.278	0:18:04	0.69995
	0:18:06	26.166	0:18:06	0.69544
	0:18:08	28.021	0:18:08	0.68851
	0:18:10	26.691	0:18:10	0.698
	0:18:12	26.984	0:18:12	0.69614
	0:18:14	28.479	0:18:14	0.69342
	0:18:16	27.789	0:18:16	0.69749
	0:18:18	27.568	0:18:18	0.69872
	0:18:20	30.3	0:18:20	0.69522
	0:18:22	28.269	0:18:22	0.64938
	0:18:24	27.145	0:18:24	0.64482

Time	MV	Time	VCO2
1:44:10	24.905	1:44:10	14.441
1:44:12	23.691	1:44:12	14.503
1:44:14	23.481	1:44:14	14.448
1:44:18	18.648	1:44:16	14.476
1:44:20	15.627	1:44:18	14.391
1:44:22	17.931	1:44:20	14.443
1:44:24	17.18	1:44:22	14.478
1:44:26	14.853	1:44:24	14.478
1:44:28	15.219	1:44:26	14.499
1:44:30	19.407	1:44:28	14.551
1:44:32	26.4	1:44:30	14.528

1:44:34	12.855	1:44:32	14.634
1:44:38	10.187	1:44:34	14.656
1:44:40	10.655	1:44:36	14.6
1:40:54	42.398	1:44:38	14.511
1:40:56	42.643	1:44:40	14.462

MVb/VCO2		G998 6 WKS	Time	MV	Time	VCO2
47.2514995						
33.7309987						
48.5176848						
49.2306214						
46.2604612						
46.6402098						
46.6615557						
46.8769738						
46.1125347						
45.4114169						
55.0983163						
51.2948219						
46.9375708						
46.4830609						
46.6260805						
46.4096426						
46.8464656						

MVb/VCO2			Time	MV	Time	VCO2
123.760497						
122.836709						
126.364021						
115.540772						
124.717894						
114.673949						
119.314031						
110.145118						
114.752011						
117.589184						
117.301269						
100.755718						
100.205149						
95.85046						
108.476612						
104.076146						
113.522471						

MVb/VCO2		G810 13 WKS	Time	MV	Time	VCO2
43.9317384						
43.0258292						
40.0723757						
41.8540297						
42.3553487						
40.4000286						
37.6251007						
40.6980291						
38.239255						
38.7623179						
41.070347						
39.8414314						
39.4550034						
43.5833261						
43.5322923						
42.0970193						
41.033967						

MVb/VCO2			Time	MV	Time	VCO2
1.72460356						
1.6335241						
1.62520764						
1.28820116						
1.08588701						
1.24150107						
1.18662799						
1.02590137						
1.0496586						
1.33372277						
1.81718062						

0.87843378
0.69507369
0.72979452
2.92178347
2.94862398
1.44910783

MVb/VCO2	G1167 10wks	Time	MV	Time
		1:12:54	37.694	1:12:54
		1:12:56	21.864	1:12:56
		1:12:58	28.712	1:12:58
		1:13:00	29.03	1:13:00
		1:13:02	27.005	1:13:02
		1:13:04	25.938	1:13:04
		1:13:06	25.063	1:13:06
		1:13:08	26.361	1:13:08
		1:13:10	33.478	1:13:10
		1:13:12	32.949	1:13:12
		1:13:14	27.03	1:13:14
		1:13:16	25.183	1:13:16
		1:13:18	27.197	1:13:18
		1:13:20	24.247	1:13:20
		1:13:22	27.104	1:13:22
		1:13:24	24.605	1:13:24

MVb/VCO2	Time	MV	Time
	1:46:38	168.71	1:46:38
	1:46:40	175.63	1:46:40
	1:46:42	179.98	1:46:42
	1:46:44	180.5	1:46:44
	1:46:46	182.68	1:46:46
	1:46:48	198.64	1:46:48
	1:46:50	177.77	1:46:50
	1:46:52	181.56	1:46:52
	1:46:54	177.03	1:46:54
	1:46:56	165.69	1:46:56
	1:46:58	182.87	1:46:58
	1:47:00	185.38	1:47:00
	1:47:02	170.66	1:47:02
	1:47:04	194.18	1:47:04
	1:47:06	170.64	1:47:06
	1:47:08	165.27	1:47:08

MVb/VCO2	G1213 9wks	Time	MV	Time
		1:09:34	28.323	1:09:34
		1:09:36	25.766	1:09:36
		1:09:38	25.353	1:09:38
		1:09:40	33.708	1:09:40
		1:09:42	26.883	1:09:42
		1:09:44	27.815	1:09:44
		1:09:46	26.606	1:09:46
		1:09:48	26.081	1:09:48
		1:09:50	32.359	1:09:50
		1:09:52	24.82	1:09:52
		1:09:54	26.131	1:09:54
		1:09:56	25.131	1:09:56
		1:09:58	26.397	1:09:58
		1:10:00	28.767	1:10:00
		1:10:02	25.126	1:10:02
		1:10:04	28.427	1:10:04

MVb/VCO2	Time	MV	Time
	1:55:04	24.181	1:54:34
	1:55:06	27.754	1:54:36
	1:55:08	24.161	1:54:38
	1:55:10	27.219	1:54:40
	1:55:12	24.654	1:54:42
	1:55:14	28.51	1:54:44
	1:55:16	25.022	1:54:46
	1:55:18	22.417	1:54:48
	1:55:20	21.193	1:54:50
	1:55:22	19.117	1:54:52
	1:55:24	22.053	1:54:54

1:55:26	22.249	1:54:56
1:55:28	23.53	1:54:58
1:55:30	24.201	1:55:00
1:55:32	24.878	1:55:02
1:55:34	25.202	1:55:04

VCO2	MVb/VCO2	G1168 13	Time	MV
0.83277	45.2633981		1:01:54	23.05
0.82992	26.3447079		1:01:56	23.263
0.82412	34.8395865		1:01:58	24.62
0.80466	36.0773494		1:02:00	23.672
0.79783	33.8480629		1:02:02	22.11
0.79515	32.6202603		1:02:04	22.97
0.79171	31.6567935		1:02:06	24.82
0.79273	33.2534406		1:02:08	25.393
0.79671	42.0203085		1:02:10	32.647
0.78396	42.02893		1:02:12	21.477
0.78897	34.2598578		1:02:14	22.696
0.79241	31.7802653		1:02:16	25.297
0.79039	34.4095953		1:02:18	27.482
0.79257	30.5928814		1:02:20	31.29
0.78983	34.3162453		1:02:22	23.449
0.78711	31.2599256		1:02:24	25.682
	34.6607255			

VCO2	MVb/VCO2	Time	MV
0.88909	189.755818	1:34:54	182.1
1.0145	173.119763	1:34:56	183.37
1.1123	161.808865	1:34:58	171.88
1.1716	154.06282	1:35:00	206.42
1.1721	155.857009	1:35:02	170.84
1.2586	157.826156	1:35:04	190.1
1.274	139.536892	1:35:06	170.65
1.2912	140.613383	1:35:08	183.26
1.2838	137.895311	1:35:10	178.29
1.2965	127.797917	1:35:12	202.73
1.3328	137.207383	1:35:14	201.05
1.3515	137.166112	1:35:16	172.55
1.3219	129.10205	1:35:18	188.43
1.31	148.229008	1:35:20	175.43
1.3897	122.789091	1:35:22	181.3
1.3636	121.201232	1:35:24	198.24
	145.873051		

VCO2	MVb/VCO2		G1214 12wks	Time	MV
0.53278	53.1607793			0:52:56	35.778
0.53619	48.0538615			0:52:58	33.72
0.53452	47.4313403			0:53:00	37.089
0.53286	63.258642			0:53:02	38.298
0.53335	50.4040499			0:53:04	30.699
0.52723	52.7568613			0:53:06	39.199
0.53303	49.914639			0:53:08	32.415
0.52511	49.6676887			0:53:10	34.173
0.53704	60.2543572			0:53:12	34.598
0.53072	46.7666566			0:53:14	35.917
0.53908	48.4733249			0:53:16	32.999
0.54841	45.8252038			0:53:18	35.675
0.53225	49.5951151			0:53:20	34.78
0.53538	53.7319287			0:53:22	47.185
0.5369	46.7982865			0:53:24	31.015
0.53614	53.0215988			0:53:26	32.92
	51.1946458				

VCO2	MVb/VCO2		Time	MV
5.3432	4.5255652		2:13:56	49.38
5.5063	5.04040826		2:13:58	47.929
5.6017	4.31315494		2:14:00	48.614
5.6139	4.84850104		2:14:02	51.591
5.6446	4.36771428		2:14:04	51.127
5.6253	5.06817414		2:14:06	53.125
5.6358	4.43983108		2:14:08	51.841
5.7101	3.92585069		2:14:10	55.63
5.7127	3.70980447		2:14:12	53.582
5.7633	3.31702323		2:14:14	51.019
5.8119	3.7944562		2:14:16	51.756

5.8277	3.81780119	2:14:18	49.432
5.8207	4.04246912	2:14:20	51.063
5.8591	4.13049786	2:14:22	52.613
5.8499	4.25272227	2:14:24	68.713
5.7911	4.35185025	2:14:26	54.8
	4.24661401		

Time	VCO2	MVb/VCO2		G1169 12 wk:	Time
1:01:54	0.65231	35.3359599			1:20:06
1:01:56	0.66349	35.0615684			1:20:08
1:01:58	0.64678	38.0654937			1:20:10
1:02:00	0.63454	37.3057648			1:20:12
1:02:02	0.62737	35.242361			1:20:14
1:02:04	0.62108	36.9839634			1:20:16
1:02:06	0.62056	39.9961325			1:20:18
1:02:08	0.6174	41.1289278			1:20:20
1:02:10	0.61561	53.031952			1:20:22
1:02:12	0.61896	34.6985266			1:20:24
1:02:14	0.61403	36.9623634			1:20:26
1:02:16	0.61613	41.0578936			1:20:28
1:02:18	0.6048	45.4398148			1:20:30
1:02:20	0.61446	50.9227614			1:20:32
1:02:22	0.59718	39.2662179			1:20:34
1:02:24	0.63548	40.4135457			1:20:36
		40.0570779			

Time	VCO2	MVb/VCO2			Time
1:34:54	1.1825	153.995772			1:35:06
1:34:56	1.2332	148.694453			1:35:08
1:34:58	1.2567	136.770908			1:35:10
1:35:00	1.2198	169.224463			1:35:12
1:35:02	1.255	136.12749			1:35:14
1:35:04	1.2234	155.386627			1:35:16
1:35:06	1.2228	139.556755			1:35:18
1:35:08	1.2618	145.236963			1:35:20
1:35:10	1.2842	138.833515			1:35:22
1:35:12	1.2893	157.240363			1:35:24
1:35:14	1.2668	158.706978			1:35:26
1:35:16	1.3269	130.039943			1:35:28
1:35:18	1.2951	145.494556			1:35:30
1:35:20	1.3013	134.811343			1:35:32
1:35:22	1.3281	136.510805			1:35:34
1:35:24	1.3058	151.814979			1:35:36
		146.15287			

Time	VCO2	MVb/VCO2		G1215 11wks	Time
0:52:56	0.62388	57.3475668			1:33:40
0:52:58	0.61358	54.9561589			1:33:42
0:53:00	0.60592	61.211051			1:33:44
0:53:02	0.61071	62.7106155			1:33:46
0:53:04	0.60811	50.4826429			1:33:48
0:53:06	0.60571	64.7157881			1:33:50
0:53:08	0.60678	53.4213389			1:33:52
0:53:10	0.60617	56.3752743			1:33:54
0:53:12	0.60428	57.2549149			1:33:56
0:53:14	0.60347	59.5174574			1:33:58
0:53:16	0.57948	56.9458825			1:34:00
0:53:18	0.58655	60.8217543			1:34:02
0:53:20	0.55204	63.002681			1:34:04
0:53:22	0.55303	85.3208687			1:34:06
0:53:24	0.56022	55.3621791			1:34:08
0:53:26	0.55238	59.5966545			1:34:10
		59.9401768			

Time	VCO2	MVb/VCO2		Time
2:13:56	0.30536	161.710768		1:51:40
2:13:58	0.38124	125.718707		1:51:42
2:14:00	0.42159	115.311084		1:51:44
2:14:02	0.47136	109.451375		1:51:46
2:14:04	0.51508	99.2603091		1:51:50
2:14:06	0.50779	104.62002		1:51:52
2:14:08	0.54218	95.6158471		1:51:54
2:14:10	0.53438	104.10195		1:51:56
2:14:12	0.5213	102.785344		1:51:58
2:14:14	0.52407	97.3514988		1:52:00
2:14:16	0.52534	98.5190543		1:52:02

2:14:18	0.53999	91.542436	1:52:04
2:14:20	0.54704	93.3441796	1:52:06
2:14:22	0.58272	90.2886463	1:52:08
2:14:24	0.59893	114.726262	1:52:10
2:14:26	0.573	95.6369983	
		106.24903	

MV	Time	VCO2	MVb/VCO2
27.767	1:20:06	0.65809	42.193317
36.552	1:20:08	0.68279	53.5332972
25.199	1:20:10	0.68002	37.0562631
23.234	1:20:12	0.67026	34.6641602
29.779	1:20:14	0.68053	43.7585411
34.856	1:20:16	0.67786	51.4206473
18.784	1:20:18	0.67284	27.9174841
30.526	1:20:20	0.65883	46.3336521
21.018	1:20:22	0.68885	30.5117224
26.425	1:20:24	0.70562	37.4493353
20.832	1:20:26	0.71178	29.2674703
23.77	1:20:28	0.68567	34.6668222
23.328	1:20:30	0.70883	32.9105709
22.713	1:20:32	0.71342	31.8367862
21.514	1:20:34	0.71199	30.2167165
24.455	1:20:36	0.71168	34.3623539
			37.3811962

MV	Time	VCO2	MVb/VCO2
217.93	1:35:06	3.0786	70.7886702
218.46	1:35:08	3.0626	71.3315484
229.87	1:35:10	3.1107	73.8965506
212.07	1:35:12	3.161	67.0895286
207.62	1:35:14	3.1914	65.0560882
205.56	1:35:16	3.1612	65.0259395
231.33	1:35:18	3.2272	71.6813337
236.9	1:35:20	3.203	73.9619107
209.3	1:35:22	3.2251	64.8972125
200.24	1:35:24	3.2339	61.9190451
193.44	1:35:26	3.2559	59.4121441
202.43	1:35:28	3.2721	61.8654687
217.64	1:35:30	3.2541	66.8817799
198.62	1:35:32	3.281	60.5364218
208.73	1:35:34	3.3017	63.2189478
218.32	1:35:36	3.3152	65.8542471
			66.4635523

MV	Time	VCO2	MVb/VCO2
26.762	1:33:40	0.51172	52.2981318
27.393	1:33:42	0.50626	54.1085608
28.855	1:33:44	0.50612	57.012171
30.207	1:33:46	0.50061	60.3403847
33.568	1:33:48	0.4998	67.1628651
33.05	1:33:50	0.51154	64.6088282
24.717	1:33:52	0.50719	48.7332163
28.028	1:33:54	0.50882	55.0843127
32.444	1:33:56	0.51078	63.5185403
29.557	1:33:58	0.51052	57.8958709
28.855	1:34:00	0.50984	56.596187
31.209	1:34:02	0.51386	60.7344413
30.026	1:34:04	0.51667	58.1144638
30.954	1:34:06	0.51124	60.5469056
29.551	1:34:08	0.51092	57.8388006
29.761	1:34:10	0.51089	58.2532443
			58.3029328

MV	Time	VCO2	MVb/VCO2
19.145	1:51:40	0.048073	398.248497
17.465	1:51:42	0.12117	144.136337
16.754	1:51:44	0.23519	71.2360219
18.984	1:51:46	0.23128	82.0823245
16.472	1:51:48	0.29961	54.9781382
16.682	1:51:50	0.35723	46.6982056
18.87	1:51:52	0.33088	57.0297389
18.059	1:51:54	0.3656	49.3955142
16.389	1:51:56	0.39607	41.3790492
36.036	1:51:58	0.40426	89.1406521
23.824	1:52:00	0.46114	51.6632693

16.061	1:52:02	0.49289	32.5853639
16.337	1:52:04	0.46105	35.4343347
24.193	1:52:06	0.47387	51.0540866
19.846	1:52:08	0.49231	40.3119985
	1:52:10	0.46629	0
			77.8358457

Metabolism
WT 8 weeks

	**G1168**	**weight**	Time	MV	Time	VCO2	MVb/VCO2	VO2
		23.4	1:28:14	37.905	1:28:14	0.80929	46.83735	1.2456
			1:28:16	39.222	1:28:16	0.80583	48.6728	1.3075
			1:28:18	42.599	1:28:18	0.79853	53.34677	1.2658
			1:28:20	41.641	1:28:20	0.80991	51.41435	1.2702
			1:28:22	39.155	1:28:22	0.81773	47.88255	1.2791
			1:28:24	45.226	1:28:24	0.82492	54.82471	1.2687
			1:28:26	43.238	1:28:26	0.81705	52.91965	1.1673
			1:28:28	40.099	1:28:28	0.81836	48.99922	1.2111
			1:28:30	39.693	1:28:30	0.82632	48.03587	1.1973
			1:28:32	44.173	1:28:32	0.81559	54.16079	1.2992
			1:28:34	39.279	1:28:34	0.79067	49.67812	1.2447
			1:28:36	38.125	1:28:36	0.8083	47.16689	1.2428
			1:28:38	39.182	1:28:38	0.80409	48.72838	1.2701
			1:28:40	39.369	1:28:40	0.81578	48.25933	1.2307
			1:28:42	39.388	1:28:42	0.82436	47.7801	1.2429
			1:28:44	37.223	1:28:44	0.82028	45.37841	1.2988
				40.34481		0.812938	49.63033	1.252613
				VO2/weigh	0.05353	32.20853		
				VCO/weigh	0.034741	MV/weigh	1.724137	49.62839

Challenge		weight	Time	MV	Time	VCO2	MVb/VCO2	VO2
		23.4	1:39:30	111.78	1:39:30	1.2532	89.19566	2.1534
			1:39:32	103.99	1:39:32	1.2674	82.04987	2.1816
			1:39:34	101.12	1:39:34	1.3121	77.0673	2.2193
			1:39:36	108.06	1:39:36	1.3704	78.85289	2.1947
			1:39:38	99.032	1:39:38	1.3615	72.73742	2.173
			1:39:40	95.54	1:39:40	1.3801	69.22687	2.2002
			1:39:42	98.573	1:39:42	1.3868	71.07946	2.2604
			1:39:44	98.067	1:39:44	1.3475	72.77699	2.2604
			1:39:46	97.072	1:39:46	1.4288	67.93953	2.2374
			1:39:48	99.444	1:39:48	1.3965	71.20945	2.3126
			1:39:50	99.672	1:39:50	1.4227	70.05834	2.2387
			1:39:52	99.115	1:39:52	1.3979	70.90278	2.2471
			1:39:54	98.89	1:39:54	1.4235	69.46962	2.3553
			1:39:56	97.16	1:39:56	1.4189	68.47558	2.2245
			1:39:58	100.04	1:39:58	1.4147	70.71464	2.2482
				100.5037		1.372133	73.45043	2.233787
				VO2/weigh	0.095461	44.99251		
				VCO/weigh	0.058638			
						MV/weigh	4.295028	73.24628

Metabolism								
mdx/utrn 6 weeks			Time	MV	Time	VCO2	MVb/VCO2	VO2
	**G1214**	**Weight**	1:31:30	28.851	1:31:30	0.66814	43.18107	0.75147
		24.2	1:31:32	25.742	1:31:32	0.68425	37.62075	0.92132
			1:31:34	25.196	1:31:34	0.67709	37.21219	0.87406
			1:31:36	25.833	1:31:36	0.6811	37.92835	0.9193
			1:31:38	23.946	1:31:38	0.67887	35.27332	1.0319
			1:31:40	22.424	1:31:40	0.68666	32.65663	0.87138
			1:31:42	22.272	1:31:42	0.71637	31.09008	0.8956
			1:31:44	22.449	1:31:44	0.74082	30.30291	1.0326
			1:31:46	22.891	1:31:46	0.74789	30.60744	0.99752
			1:31:48	23.951	1:31:48	0.7682	31.17808	0.8361
			1:31:50	22.175	1:31:50	0.78314	28.3155	0.95179
			1:31:52	21.663	1:31:52	0.80637	26.86484	1.0425
			1:31:54	22.054	1:31:54	0.81927	26.91909	1.0686
			1:31:56	23.149	1:31:56	0.82132	28.18512	1.1111
			1:31:58	27.566	1:31:58	0.8181	33.69515	1.0757
			1:32:00	16.004	1:32:00	0.85096	18.80699	1.2089
				23.51038		0.746784	31.86484	0.974365
				VO2/weigh	0.040263	24.12892		
				VCO/weigh	0.030859			
						MV/weigh	0.971503	31.48215
			Time	MV	Time	VCO2	MVb/VCO2	VO2
Challenge		24.2	1:49:30	94.444	1:49:30	0.47848	197.3834	0.48918
			1:49:32	92.919	1:49:32	0.56755	163.7195	0.65333
			1:49:34	85.426	1:49:34	0.65706	130.0125	0.64658
			1:49:36	84.105	1:49:36	0.67366	124.8478	0.7754
			1:49:38	90.545	1:49:38	0.74714	121.1888	0.85097
			1:49:40	87.544	1:49:40	0.73322	119.3966	0.87584
			1:49:42	85.833	1:49:42	0.76046	112.8698	0.88834
			1:49:44	85.587	1:49:44	0.79235	108.0167	0.90031
			1:49:46	86.759	1:49:46	0.77569	111.8475	0.98393
			1:49:48	84.194	1:49:48	0.78693	106.9905	0.91275
			1:49:50	82.763	1:49:50	0.79366	104.2802	0.93705
			1:49:52	91.302	1:49:52	0.81642	111.8321	0.93867
			1:49:54	85.552	1:49:54	0.76133	112.3718	1.0104

1:49:56	83.94	1:49:56	0.82163	102.1628	0.92591
1:49:58	109.46	1:49:58	0.8316	131.6258	0.98234
1:50:00	98.7	1:50:00	0.81549	121.0315	0.89265
	89.31706		0.738292	123.7236	0.853978
	VO2/weigh	0.035288	104.5894		
	VCO/weigh	0.030508			
			MV/weigh	3.690788	120.978

MV/VO2	RQ	G1169	weight	Time	MV	Time	VCO2
30.43112	0.64972		24.1	0:47:44	29.969	0:47:44	0.92963
29.99771	0.61985			0:47:46	29.141	0:47:46	0.93089
33.65382	0.63218			0:47:48	28.738	0:47:48	0.92167
32.78303	0.63873			0:47:50	29.096	0:47:50	0.92327
30.61137	0.64247			0:47:52	29.98	0:47:52	0.90554
35.64751	0.65858			0:47:54	25.358	0:47:54	0.89871
37.04103	0.70119			0:47:56	28.017	0:47:56	0.90914
33.10957	0.67763			0:47:58	27.963	0:47:58	0.90539
33.15209	0.69551			0:48:00	25.836	0:48:00	0.91897
34.00015	0.6296			0:48:02	28.605	0:48:02	0.89534
31.557	0.63732			0:48:04	26.265	0:48:04	0.89206
30.6767	0.65098			0:48:06	27.314	0:48:06	0.88662
30.84954	0.63535			0:48:08	26.977	0:48:08	0.88378
31.98911	0.66403			0:48:10	28.915	0:48:10	0.88606
31.6904	0.66549			0:48:12	38.261	0:48:12	0.8729
28.65953	0.63192			0:48:14	32.851	0:48:14	0.86674
32.24061	0.651909				28.95538		0.901669
					VO2/weigh	0.058233	20.63221
					VCO/weigh	0.037414	

MV/VO2	RQ		weight	Time	MV	Time	VCO2
51.90861	0.58314		24.1	1:44:00	190.35	1:44:00	1.3991
47.66685	0.58113			1:44:02	170.45	1:44:02	1.4316
45.56392	0.59144			1:44:04	181.99	1:44:04	1.3728
49.2368	0.62475			1:44:06	195.25	1:44:06	1.3606
45.57386	0.62676			1:44:08	161.48	1:44:08	1.3994
43.42333	0.62822			1:44:10	162.85	1:44:10	1.4129
43.60865	0.61563			1:44:12	190.09	1:44:12	1.4399
43.3848	0.59618			1:44:14	168.33	1:44:14	1.3993
43.38607	0.63885			1:44:16	179.3	1:44:16	1.4222
43.00095	0.60436			1:44:18	194.06	1:44:18	1.3968
44.52227	0.63637			1:44:20	166.51	1:44:20	1.4159
44.10796	0.62179			1:44:22	177.44	1:44:22	1.4677
41.98616	0.60446			1:44:24	195.86	1:44:24	1.4204
43.67723	0.63859			1:44:26	200.47	1:44:26	1.4537
44.49782	0.63066			1:44:28	188.71	1:44:28	1.4323
45.03635	0.614822			1:44:30	167.58	1:44:30	1.3322
					180.67		1.4098
					VO2/weigh	0.072523	103.3692
					VCO/weigh	0.058498	

MV/VO2	RQ						
38.39275	0.91338	**G1215**		Time	MV	Time	VCO2
27.94035	0.74487		**weight**	1:08:14	30.41	1:08:14	0.71172
28.8264	0.80012		23.3	1:08:16	28.123	1:08:16	0.70149
28.10073	0.74777			1:08:18	26.744	1:08:18	0.72027
23.20574	0.65861			1:08:20	26.202	1:08:20	0.71073
25.73389	0.79158			1:08:22	27.422	1:08:22	0.71714
24.86824	0.81415			1:08:24	30.721	1:08:24	0.70628
21.74027	0.71896			1:08:26	30.331	1:08:26	0.70637
22.94791	0.75182			1:08:28	27.798	1:08:28	0.72854
28.64609	0.92693			1:08:30	29.11	1:08:30	0.72179
23.29821	0.82722			1:08:32	28.254	1:08:32	0.70955
20.77986	0.77668			1:08:34	27.18	1:08:34	0.69699
20.63822	0.77175			1:08:36	38.137	1:08:36	0.68731
20.83431	0.7436			1:08:38	33.067	1:08:38	0.69191
25.6261	0.77663			1:08:40	30.152	1:08:40	0.68508
13.23848	0.70393			1:08:42	30.465	1:08:42	0.68077
24.6761	0.77925			1:08:44	26.273	1:08:44	0.67088
					29.39931		0.702926
					VO2/weigl	0.053266	23.68835
					VCO/weigl	0.030169	

MV/VO2	RQ						
193.0659	0.99496			Time	MV	Time	VCO2
142.2237	0.89934		weight	1:50:14	49.733	1:50:14	0.54729
132.1198	1.0327		23.3	1:50:16	57.893	1:50:16	0.5947
108.4666	0.8928			1:50:18	55.333	1:50:18	0.62533
106.4021	0.88701			1:50:20	53.866	1:50:20	0.56537
99.95433	0.84446			1:50:22	54.077	1:50:22	0.60363
96.62179	0.86087			1:50:24	56.34	1:50:24	0.58717
95.06392	0.90281			1:50:26	54.42	1:50:26	0.62061
88.17599	0.79133			1:50:28	56.248	1:50:28	0.62507
92.24213	0.87647			1:50:30	54.598	1:50:30	0.58969
88.32293	0.86007			1:50:32	54.013	1:50:32	0.65696
97.26741	0.89414			1:50:34	54.493	1:50:34	0.66182
84.67142	0.75574			1:50:36	53.225	1:50:36	0.75656

90.65676	0.89457	1:50:38	54.782	1:50:38		0.71746
111.4278	0.85135	1:50:40	54.359	1:50:40		0.6883
110.5697	0.91356	1:50:42	56.449	1:50:42		0.72473
108.5783	0.884511	1:50:44	51.771	1:50:44		0.70035
			54.475			0.641565
			VO2/weigh		0.030605	76.39335
			VCO/weigh		0.027535	

MVb/VCO2	VO2	MV/VO2	RQ		G1368	weight
32.23756	1.3258	22.60447	0.70119			18.4
31.30445	1.4439	20.18215	0.64577			
31.18036	1.4306	20.08807	0.64481			
31.51407	1.4287	20.36537	0.64973			
33.10732	1.4201	21.11119	0.63779			
28.216	1.3357	18.9848	0.67537			
30.81704	1.4312	19.57588	0.63812			
30.88503	1.4176	19.72559	0.64137			
28.11408	1.4142	18.26899	0.6502			
31.94876	1.4221	20.11462	0.6299			
29.44309	1.3801	19.03123	0.65118			
30.80688	1.441	18.95489	0.61785			
30.52456	1.3829	19.50756	0.64021			
32.63323	1.3987	20.67277	0.6356			
43.83205	1.432	26.71858	0.61111			
37.90179	1.3499	24.33588	0.64326			
32.15414	1.403406	20.64013	0.644591			

MV/weigh	1.201468	32.11307

MVb/VCO2	VO2	MV/VO2	RQ		weight
136.0517	1.7459	109.0269	0.80626		18.4
119.0626	1.7754	96.00653	0.80791		
132.5685	1.7601	103.3975	0.78223		
143.5029	1.7884	109.1758	0.76265		
115.3923	1.7537	92.0796	0.8		
115.2594	1.7315	94.0514	0.81642		
132.0161	1.6325	116.441	0.88677		
120.2959	1.7723	94.97828	0.79059		
126.0723	1.6735	107.1407	0.85159		
138.9318	1.7525	110.7332	0.79769		
117.6001	1.8207	91.45384	0.78103		
120.8966	1.835	96.69755	0.80383		
137.8907	1.7334	112.9918	0.81943		
137.9033	1.6976	118.0902	0.85818		
131.7531	1.709	110.4213	0.83886		
125.7919	1.7835	93.96131	0.74696		
128.1868	1.747813	103.5404	0.8094		

MV/weigh	7.49668	128.1529

G1352 weight

MVb/VCO2	VO2	MV/VO2	RQ	weight
				17
42.72748	1.0586	28.72662	0.67235	
40.09038	1.1825	23.78266	0.59639	
37.13052	1.2824	20.85465	0.56574	
36.86632	1.3163	19.9058	0.54154	
38.238	1.2077	22.70597	0.59771	
43.49691	1.3664	22.48317	0.52025	
42.93925	1.2084	25.10013	0.58649	
38.15576	1.3762	20.1991	0.5301	
40.33029	1.2384	23.50614	0.58504	
39.8196	1.351	20.9134	0.5299	
38.99626	1.2764	21.29427	0.54895	
55.48733	1.2976	29.39041	0.53094	
47.7909	1.2378	26.71433	0.5612	
44.01238	1.1775	25.60679	0.58552	
44.7508	1.1727	25.97851	0.58562	
39.162	1.1075	23.7228	0.60925	
41.87464	1.241088	23.8053	0.571687	

MV/weigh	1.261773	41.82418

MVb/VCO2	VO2	MV/VO2	RQ	weight
90.87138	0.75396	65.96239	0.72589	17
97.34824	0.64437	89.84434	0.95272	
88.48608	0.72064	76.78314	0.87668	
95.27566	0.68451	78.69279	0.83774	
89.58634	0.63989	84.50984	0.99043	
95.95177	0.70728	79.65728	0.83245	
87.68792	0.73406	74.13563	0.84757	
89.98672	0.71873	78.26026	0.89389	
92.58763	0.76258	71.59642	0.78506	
82.21657	0.64797	83.35725	1.0268	
82.3381	0.67409	80.83935	0.984	
70.35133	0.67839	78.45782	1.1218	

76.35548	0.79122	69.23738	0.91321
78.97574	0.76776	70.80207	0.92066
77.8897	0.7638	73.90547	0.95489
73.92161	0.72012	71.89218	0.97212
85.61439	0.713086	76.74585	0.914744

MV/weigh	2.337983	84.90956

Time	MV	Time	VCO2	MVb/VCO2	VO2	MV/VO2	RQ
1:00:10	19.571	1:00:10	0.71702	27.29492	1.2862	15.21614	0.55909
1:00:12	20.051	1:00:12	0.72597	27.6196	1.2757	15.71765	0.57283
1:00:14	20.604	1:00:14	0.73074	28.19608	1.3153	15.66487	0.55671
1:00:16	19.493	1:00:16	0.7309	26.66986	1.2724	15.31987	0.57725
1:00:18	18.401	1:00:18	0.7249	25.38419	1.3338	13.79592	0.54362
1:00:20	20.562	1:00:20	0.72051	28.53812	1.2181	16.88039	0.59479
1:00:22	19.712	1:00:22	0.72545	27.1721	1.2628	15.60976	0.57612
1:00:24	18.059	1:00:24	0.71523	25.24922	1.3265	13.61402	0.5427
1:00:26	19.021	1:00:26	0.70931	26.8162	1.2823	14.8335	0.5548
1:00:28	19.064	1:00:28	0.68242	27.93588	1.2586	15.14699	0.54525
1:00:30	18.241	1:00:30	0.68875	26.48421	1.2435	14.66908	0.55769
1:00:32	18.87	1:00:32	0.68275	27.63823	1.1585	16.2883	0.59056
1:00:34	21.277	1:00:34	0.68045	31.26901	1.272	16.7272	0.53745
1:00:36	17.935	1:00:36	0.68428	26.21003	1.1876	15.10189	0.57641
1:00:38	19.709	1:00:38	0.67261	29.30227	1.1742	16.78505	0.57386
1:00:40	18.937	1:00:40	0.67949	27.86943	1.0856	17.44381	0.6259
	19.34419		0.704424	27.47808	1.247069	15.5509	0.567814
VO2/weigh	0.067775	15.51172					
VCO/weigh	0.038284						
			MV/weigh	1.051315	27.46101		

Time	MV	Time	VCO2	MVb/VCO2	VO2	MV/VO2	RQ
1:22:40	146.41	1:22:40	1.005	145.6816	1.4282	102.5137	0.70617
1:22:42	126.64	1:22:42	1.0356	122.2866	1.3906	91.0686	0.75031
1:22:44	127.78	1:22:44	1.0557	121.0382	1.4887	85.83328	0.71353
1:22:46	121.7	1:22:46	1.09	111.6514	1.4415	84.42595	0.75821
1:22:48	123.42	1:22:48	1.0934	112.8773	1.4526	84.96489	0.75671
1:22:50	128.54	1:22:50	1.0671	120.4573	1.4196	90.54663	0.75182
1:22:52	147.64	1:22:52	1.0602	139.2567	1.4866	99.31387	0.71401
1:22:54	131.41	1:22:54	1.058	124.206	1.4657	89.65682	0.72256
1:22:56	129	1:22:56	1.0573	122.0089	1.4494	89.00235	0.731
1:22:58	143.82	1:22:58	1.0882	132.1632	1.3967	102.9713	0.78215
1:23:00	130.12	1:23:00	1.0861	119.8048	1.4832	87.72923	0.73453
1:23:02	131.27	1:23:02	1.0815	121.3777	1.4418	91.04591	0.75113
1:23:04	125.74	1:23:04	1.085	115.8894	1.4382	87.42873	0.75474
1:23:06	124.77	1:23:06	1.0674	116.8915	1.43	87.25175	0.74633
1:23:08	134.28	1:23:08	1.0553	127.2434	1.4938	89.89155	0.70759
1:23:10	121.63	1:23:10	1.1466	106.0788	1.4609	83.2569	0.78486
	130.8856		1.070775	122.4321	1.447969	90.43134	0.741603

VO2/weigh	0.078694	90.39258	
VCO/weigh	0.058194		
		MV/weigh	7.113349	122.2345

Time	MV	Time	VCO2	MVb/VCO2	VO2	MV/VO2	RQ
0:21:54	21.602	0:21:54	0.57976	37.26025	1.1211	19.26858	0.51713
0:21:56	24.676	0:21:56	0.57218	43.12629	0.9484	26.01856	0.61334
0:21:58	30.159	0:21:58	0.56856	53.04453	0.99266	30.382	0.58221
0:22:00	24.111	0:22:00	0.55617	43.35185	1.0936	22.04737	0.51591
0:22:02	21.833	0:22:02	0.56416	38.70001	1.033	21.13553	0.55414
0:22:04	23.997	0:22:04	0.57638	41.63399	0.9751	24.60978	0.59958
0:22:06	23.972	0:22:06	0.5833	41.09721	0.99208	24.16337	0.58895
0:22:08	22.831	0:22:08	0.57142	39.95485	0.91813	24.86685	0.63322
0:22:10	21.387	0:22:10	0.57601	37.12956	0.97454	21.94574	0.59247
0:22:12	22.389	0:22:12	0.57377	39.02086	0.99214	22.56637	0.58228
0:22:14	29.017	0:22:14	0.58353	49.72666	1.0046	28.88413	0.59125
0:22:16	22.775	0:22:16	0.57695	39.47482	0.96395	23.62674	0.60947
0:22:18	26.487	0:22:18	0.57561	46.01553	1.0366	25.5518	0.56165
0:22:20	22.008	0:22:20	0.57809	38.0702	1.0046	21.90723	0.58915
0:22:22	23.022	0:22:22	0.57125	40.30109	1.043	22.07287	0.55193
0:22:24	22.661	0:22:24	0.57206	39.61298	0.92716	24.4413	0.62362
	23.93294		0.5737	41.72004	1.001291	23.96801	0.581644

VO2/weigh	0.058899	23.90207		
VCO/weigh	0.033747			
		MV/weigh	1.40782	41.71682

Time	MV	Time	VCO2	MVb/VCO2	VO2	MV/VO2	RQ
1:25:40	77.765	1:25:40	0.72185	107.7301	0.8908	87.29793	0.81625
1:25:42	72.024	1:25:42	0.6696	107.5627	0.89704	80.29073	0.74646
1:25:44	70.713	1:25:44	0.73451	96.27234	0.90591	78.05742	0.82078
1:25:46	74.301	1:25:46	0.71638	103.7173	0.9432	78.77545	0.76876
1:25:48	73.533	1:25:48	0.7121	103.2622	0.95738	76.80649	0.74467
1:25:50	72.294	1:25:50	0.70735	102.204	0.96034	75.27959	0.73717
1:25:52	69.574	1:25:52	0.75345	92.34057	0.95792	72.63028	0.79439
1:25:54	71.626	1:25:54	0.75129	95.33735	0.96579	74.16312	0.78115
1:25:56	83.646	1:25:56	0.76461	109.3969	0.92597	90.33338	0.82637
1:25:58	82.385	1:25:58	0.76272	108.0147	0.90403	91.13083	0.85388
1:26:00	85.069	1:26:00	0.76986	110.4993	0.91988	92.47837	0.83968
1:26:02	76.659	1:26:02	0.73446	104.3746	0.93057	82.37854	0.79283

1:26:04	69.38	1:26:04	0.75749	91.59197	0.93829	73.94302	0.80895
1:26:06	69.238	1:26:06	0.74861	92.48875	0.92712	74.68073	0.80835
1:26:08	76.577	1:26:08	0.77655	98.61181	0.94634	80.91912	0.82558
	74.9856		0.738722	101.5603	0.931372	80.611	0.797685
	VO2/weigh	0.054787	80.5109				
	VCO/weigh	0.043454					
				MV/weigh	4.410918	101.5072	

	Time	MV	Time

Time	MV	Time

VCO2	MVb/VCO2

VCO2	MVb/VCO2

Metabolism								
WT 8 weeks		Baseline						
**G998**	**weight**	Time	MV	Time	VCO2	MVb/VCO2	VO2	MV/VO2
	22.3	1:17:58	29.383	1:17:58	1.0649	27.59226	1.5924	18.45202
		1:18:00	29.42	1:18:00	1.0654	27.61404	1.6935	17.37231
		1:18:02	30.613	1:18:02	1.0693	28.62901	1.6401	18.66533
		1:18:04	28.145	1:18:04	1.064	26.45207	1.6572	16.98347
		1:18:06	27.01	1:18:06	1.0599	25.48354	1.7271	15.63893
		1:18:08	27.347	1:18:08	1.0441	26.19194	1.717	15.9272
		1:18:10	27.199	1:18:10	1.0457	26.01033	1.6576	16.40866
		1:18:12	23.856	1:18:12	1.0583	22.54181	1.6595	14.37541
		1:18:14	30.222	1:18:14	1.0355	29.1859	1.6641	18.16117
		1:18:16	27.342	1:18:16	1.0437	26.19718	1.7377	15.73459
		1:18:18	25.359	1:18:18	1.0586	23.95522	1.66	15.27651
		1:18:20	23.486	1:18:20	1.0497	22.37401	1.7145	13.69845
		1:18:22	27.218	1:18:22	1.0459	26.02352	1.7199	15.82534
		1:18:24	28.578	1:18:24	1.0499	27.21974	1.6673	17.14029
		1:18:26	26.66	1:18:26	1.0513	25.35908	1.7316	15.39617
			27.45587		1.053747	26.05531	1.682633	16.33706
		VO2/weigh	0.075454	16.3172				
		VCO/weigh	0.047253		MV/weigh	1.231205	26.05547	
Challenge	weight	Time	MV	Time	VCO2	MVb/VCO2	VO2	MV/VO2
	22.3	1:44:12	119.75	1:44:12	1.0914	109.7215	1.4698	81.47367
		1:44:14	125.17	1:44:14	1.0411	120.2286	1.6143	77.53825
		1:44:16	123.83	1:44:16	1.0823	114.4137	1.6369	75.64909
		1:44:18	119.24	1:44:18	1.0878	109.6157	1.6895	70.57709
		1:44:20	123.62	1:44:20	1.0251	120.5931	1.5896	77.76799
		1:44:22	132.72	1:44:22	1.012	131.1462	1.5009	88.42694
		1:44:24	152.56	1:44:24	0.97261	156.8563	1.501	101.6389
		1:44:26	163.45	1:44:26	0.96498	169.3817	1.4304	114.2687
		1:44:28	151.15	1:44:28	0.94813	159.4191	1.4178	106.6088
		1:44:30	155.27	1:44:30	0.89806	172.8949	1.4347	108.2247
		1:44:32	138.46	1:44:32	0.94744	146.1412	1.4178	97.65834
		1:44:34	119.71	1:44:34	0.9271	129.1231	1.3973	85.67237
		1:44:36	134.1	1:44:36	1.0291	130.308	1.4641	91.5921
		1:44:38	125.92	1:44:38	1.046	120.3824	1.5652	80.44978
		1:44:40	128.49	1:44:40	1.0777	119.2261	1.5613	82.2968
		1:44:42	137.77	1:44:42	1.0546	130.6372	1.6432	83.8425
			134.4506		1.012839	133.7556	1.520863	88.98038
			VO2/weigh	0.0682	88.40419			
			VCO/weigh	0.045419				

MV/weigh	6.029176	132.7463

Metabolism

mdx/utrn 6 weeks		Baseline						
**G810**	**Weight**	Time	MV	Time	VCO2	MVb/VCO2	VO2	MV/VO2
	17.7	1:43:36	35.446	1:43:36	0.62252	56.93954	1.2002	29.53341
		1:43:38	29.244	1:43:38	0.63014	46.40873	1.3533	21.6094
		1:43:40	31.471	1:43:40	0.63274	49.73765	1.3121	23.98521
		1:43:42	33.137	1:43:42	0.62824	52.74577	1.2211	27.13701
		1:43:44	33.28	1:43:44	0.62877	52.92873	1.1853	28.07728
		1:43:46	32.818	1:43:46	0.61999	52.93311	1.2233	26.82743
		1:43:48	31.998	1:43:48	0.6263	51.09053	1.3199	24.24275
		1:43:50	29.297	1:43:50	0.62339	46.99626	1.3046	22.45669
		1:43:52	28.111	1:43:52	0.63144	44.51888	1.2471	22.5411
		1:43:54	24.174	1:43:54	0.62236	38.84247	1.1534	20.9589
		1:43:56	30.019	1:43:56	0.62487	48.04039	1.2699	23.63887
		1:43:58	29.072	1:43:58	0.62916	46.20764	1.3321	21.82419
		1:44:00	41.778	1:44:00	0.62689	66.64327	1.3431	31.10565
		1:44:02	38.641	1:44:02	0.61898	62.4269	1.3667	28.27321
		1:44:04	21.909	1:44:04	0.62625	34.98443	1.2514	17.50759
		1:44:06	20.03	1:44:06	0.62626	31.98352	1.4507	13.80713
			30.65156		0.626144	48.96424	1.283388	23.97036
			VO2/weigh	0.072508	23.88333			
			VCO/weigh	0.035375				
					MV/weigh	1.731727	48.95292	

Challenge	Weight	Time	MV	Time	VCO2	MVb/VCO2	VO2	MV/VO2
	17.7	1:52:36	88.469	1:52:36	0.56715	155.9887	0.87647	100.9379
		1:52:38	73.515	1:52:38	0.62165	118.2579	0.90193	81.50854
		1:52:40	78.475	1:52:40	0.6463	121.4219	0.8733	89.8603
		1:52:42	77.466	1:52:42	0.59654	129.8589	0.88931	87.10798
		1:52:44	75.288	1:52:44	0.66417	113.3565	0.89499	84.12161
		1:52:46	85.091	1:52:46	0.66069	128.7911	0.86634	98.21894
		1:52:48	87.438	1:52:48	0.67251	130.0174	0.85165	102.6689
		1:52:50	85.244	1:52:50	0.65137	130.8688	0.93661	91.01334
		1:52:52	83.861	1:52:52	0.6537	128.2867	0.90998	92.15697
		1:52:54	81.244	1:52:54	0.64135	126.6765	0.91141	89.141
		1:52:56	82.669	1:52:56	0.74221	111.3822	0.92884	89.00241
		1:52:58	84.691	1:52:58	0.69116	122.5346	0.89522	94.60356

1:53:00	86.345	1:53:00	0.70339	122.7555	0.84149	102.6097
1:53:02	77.297	1:53:02	0.7071	109.3155	0.92067	83.95734
1:53:04	75.066	1:53:04	0.69231	108.4283	0.86892	86.39
1:53:06	76.879	1:53:06	0.72303	106.3289	1.0257	74.95272
	81.18988		0.664664	122.7668	0.899552	90.5157
	VO2/weigh	0.050822	90.25591			
	VCO/weigh	0.037552				
			MV/weigh	4.586999	122.1517	

RQ		G1168	weight	Time	MV	Time	VCO2	MVb/VCO2
0.6698			24.3	1:12:40	37.137	1:20:16	0.73489	50.53409
0.62991				1:12:42	36.537	1:20:18	0.73756	49.53766
0.65391				1:12:44	36.013	1:20:20	0.73797	48.80009
0.64314				1:12:46	36.104	1:20:22	0.73438	49.16256
0.61443				1:12:48	36.203	1:20:24	0.73495	49.25913
0.61038				1:12:50	37.13	1:20:26	0.73173	50.74276
0.63164				1:12:52	38.868	1:20:28	0.72434	53.65988
0.63808				1:12:54	38.17	1:20:30	0.71246	53.57494
0.62342				1:12:56	36.689	1:20:32	0.73354	50.01636
0.60134				1:12:58	36.126	1:20:34	0.73124	49.40375
0.63884				1:13:00	33.725	1:20:36	0.70773	47.65235
0.61276				1:13:02	36.143	1:20:38	0.68301	52.91723
0.61059				1:13:04	36.812	1:20:40	0.6944	53.01267
0.63028				1:13:06	34.679	1:20:42	0.71827	48.28129
0.60857				1:13:08	35.552	1:20:44	0.71972	49.39699
0.627806				1:13:10	36.801	1:20:46	0.71022	51.81634
					36.41806		0.721651	50.48551
					VO2/weigh	0.04833	31.0096	
					VCO/weigh	0.029698	MV/weigh	1.498686

RQ		weight	Time	MV	Time	VCO2	MVb/VCO2
0.74254		24.3	1:50:10	188.75	1:50:10	0.96534	195.527
0.64513			1:50:12	160.29	1:50:12	0.98054	163.4711
0.66218			1:50:14	182.82	1:50:14	1.048	174.4466
0.64535			1:50:16	228.96	1:50:16	1.0491	218.2442
0.64675			1:50:18	194.17	1:50:18	1.1175	173.7539
0.67971			1:50:20	184	1:50:20	1.1518	159.75
0.64791			1:50:22	213.31	1:50:22	1.2098	176.3184
0.67774			1:50:24	193.54	1:50:24	1.1596	166.9024
0.67091			1:50:26	187.69	1:50:26	1.1747	159.777
0.62686			1:50:28	188.27	1:50:28	1.1662	161.4389
0.67124			1:50:30	152.7	1:50:30	1.1838	128.9914
0.66433			1:50:32	158.79	1:50:32	1.2108	131.1447
0.7062			1:50:34	189.99	1:50:34	1.2794	148.4993
0.6688			1:50:36	197.62	1:50:36	1.2688	155.7535
0.69369			1:50:38	169.49	1:50:38	1.2543	135.1272
0.64521				186.026		1.147979	163.2764
0.668409				VO2/weigh	0.056906	134.5278	
				VCO/weigh	0.047242		
						MV/weigh	7.655391

RQ			Time	MV	Time	VCO2	MVb/VCO2
RQ	**G1214**	**Weight**	0:32:16	41.235	0:32:16	0.82328	50.08624
0.52405		21.9	0:32:18	42.607	0:32:18	0.81244	52.44326
0.47118			0:32:20	37.855	0:32:20	0.79515	47.60737
0.48272			0:32:22	38.077	0:32:22	0.81567	46.68187
0.52642			0:32:24	41.046	0:32:24	0.843	48.69039
0.53156			0:32:26	37.519	0:32:26	0.86973	43.13868
0.50773			0:32:28	37.747	0:32:28	0.88101	42.84514
0.47812			0:32:30	40.878	0:32:30	0.90776	45.03173
0.48014			0:32:32	43.275	0:32:32	0.93391	46.33744
0.50856			0:32:34	40.247	0:32:34	0.93768	42.92189
0.54473			0:32:36	35.712	0:32:36	0.92813	38.47737
0.50358			0:32:38	40.121	0:32:38	0.9152	43.83851
0.47576			0:32:40	35.09	0:32:40	0.89702	39.11841
0.46704			0:32:42	38.29	0:32:42	0.89167	42.9419
0.4546			0:32:44	37.357	0:32:44	0.8921	41.87535
0.50317			0:32:46	50.358	0:32:46	0.87905	57.28684
0.43231				39.83838		0.876425	45.58265
0.493229				VO2/weigh	0.071325	25.50431	
				VCO/weigh	0.040019		
						MV/weigh	1.819104

RQ		Time	MV	Time	VCO2	MVb/VCO2
RQ	21.9	1:43:32	80.457	1:43:32	0.76603	105.0311
0.65643		1:43:34	85.97	1:43:34	0.75965	113.1705
0.6906		1:43:36	84.681	1:43:36	0.7657	110.5929
0.74877		1:43:38	88.086	1:43:38	0.77525	113.6227
0.67864		1:43:40	87.797	1:43:40	0.80592	108.9401
0.7436		1:43:42	88.662	1:43:42	0.76469	115.945
0.77033		1:43:44	86.284	1:43:44	0.80142	107.6639
0.79101		1:43:46	85.558	1:43:46	0.80617	106.129
0.69972		1:43:48	91.698	1:43:48	0.78178	117.2939
0.72021		1:43:50	102.42	1:43:50	0.81063	126.3462
0.70634		1:43:52	95.747	1:43:52	0.82018	116.739
0.8012		1:43:54	96.26	1:43:54	0.83189	115.7124
0.77751		1:43:56	95.975	1:43:56	0.82761	115.9665

0.84348		1:43:58	96.35	1:43:58	0.82074	117.3941
0.77482		1:44:00	95.11	1:44:00	0.82904	114.7231
0.80987			90.737		0.79778	113.6847
0.70533			VO2/weigh	0.04516	91.74578	
0.744866			VCO/weigh	0.036428		
					MV/weigh	4.143242

VO2	MV/VO2	RQ	**G1169**	**weight**	Time	MV	Time
1.0773	34.47229	0.82291		23.7	1:13:24	23.803	1:13:24
1.1038	33.10111	0.80189			1:13:26	25.189	1:13:26
1.0865	33.14588	0.82493			1:13:28	24.418	1:13:28
1.0883	33.17468	0.80821			1:13:30	24.692	1:13:30
1.2861	28.14944	0.68766			1:13:32	23.3	1:13:32
1.1979	30.99591	0.72708			1:13:34	24.93	1:13:34
1.2484	31.13425	0.69689			1:13:36	35.493	1:13:36
1.243	30.70796	0.7234			1:13:38	27.938	1:13:38
1.2701	28.8867	0.68891			1:13:40	20.076	1:13:40
1.1506	31.39753	0.75213			1:13:42	25.701	1:13:42
1.1645	28.96093	0.74296			1:13:44	25.915	1:13:44
1.0412	34.71283	0.84648			1:13:46	45.527	1:13:46
1.15	32.01043	0.75287			1:13:48	18.878	1:13:48
1.1518	30.10853	0.75194			1:13:50	25.347	1:13:50
1.2903	27.55328	0.66825			1:13:52	26.327	1:13:52
1.2408	29.65909	0.70152			1:13:54	23.581	1:13:54
1.174413	31.13568	0.749877				26.31969	
						VO2/weigh	0.056607
50.46495						VCO/weigh	0.038813

VO2	MV/VO2	RQ		weight	Time	MV	Time
1.1202	168.4967	0.87289		23.7	1:50:54	163.37	1:50:54
1.2343	129.8631	0.79769			1:50:56	167.54	1:50:56
1.1854	154.2264	0.88651			1:50:58	153.74	1:50:58
1.3038	175.6098	0.81787			1:51:00	138.17	1:51:00
1.2911	150.3911	0.86908			1:51:02	134.71	1:51:02
1.3671	134.5915	0.84462			1:51:04	139.94	1:51:04
1.4382	148.3173	0.85075			1:51:06	175.04	1:51:06
1.4061	137.6431	0.82492			1:51:08	153	1:51:08
1.4096	133.1512	0.83891			1:51:10	163.8	1:51:10
1.4091	133.6101	0.83055			1:51:12	154.55	1:51:12
1.4444	105.7186	0.82506			1:51:14	176.04	1:51:14
1.5833	100.2905	0.76942			1:51:16	177.76	1:51:16
1.4528	130.7751	0.8842			1:51:18	182.5	1:51:18
1.5532	127.2341	0.81689			1:51:20	151.67	1:51:20
1.5435	109.8089	0.81323			1:51:22	131	1:51:22
1.382807	135.9818	0.836173				157.522	
						VO2/weigh	0.047039
						VCO/weigh	0.037347
162.0466							

VO2	MV/VO2	RQ			Time	MV	Time
1.4666	28.11605	0.56202	**G1215**	**weight**	0:55:30	34.598	0:55:30
1.4651	29.08129	0.55479		17.9	0:55:32	36.206	0:55:32
1.4061	26.92198	0.56578			0:55:34	50.466	0:55:34
1.5522	24.53099	0.52807			0:55:36	38.143	0:55:36
1.4409	28.48636	0.58942			0:55:38	40.424	0:55:38
1.5118	24.81744	0.57557			0:55:40	35.576	0:55:40
1.6622	22.70906	0.53117			0:55:42	34.395	0:55:42
1.6388	24.94386	0.55694			0:55:44	33.066	0:55:44
1.6144	26.80562	0.58076			0:55:46	32.959	0:55:46
1.6667	24.14772	0.56967			0:55:48	30.697	0:55:48
1.5909	22.44767	0.58555			0:55:50	31.515	0:55:50
1.5781	25.42361	0.58354			0:55:52	29.899	0:55:52
1.6244	21.60182	0.55373			0:55:54	31.925	0:55:54
1.5206	25.18085	0.58684			0:55:56	34.598	0:55:56
1.5341	24.35109	0.58294			0:55:58	32.753	0:55:58
1.7195	29.28642	0.51122			0:56:00	30.771	0:56:00
1.562025	25.55324	0.563626				34.87444	
						VO2/weigh	0.069642
						VCO/weigh	0.039592
45.45554							

VO2	MV/VO2	RQ			Time	MV	Time
0.87768	91.67008	0.87967		weight	1:44:16	60.108	1:44:16
0.94511	90.96296	0.80958		17.9	1:44:18	55.218	1:44:18
0.89161	94.97538	0.86746			1:44:20	57.643	1:44:20
0.97016	90.79533	0.80753			1:44:22	51.14	1:44:22
1.0054	87.32544	0.81018			1:44:24	55.416	1:44:24
1.0488	84.53661	0.73715			1:44:26	55.416	1:44:26
1.0018	86.12897	0.81321			1:44:28	55.117	1:44:28
0.92841	92.15541	0.86863			1:44:30	54.933	1:44:30
1.0172	90.14746	0.77			1:44:32	53.009	1:44:32
1.0558	97.00701	0.76796			1:44:34	55.711	1:44:34
0.99586	96.14504	0.8347			1:44:36	68.521	1:44:36
1.1043	87.16834	0.75538			1:44:38	56.574	1:44:38
0.9909	96.85639	0.8406			1:44:40	53.004	1:44:40

1.0225	94.22983	0.81287	1:44:42	59.367	1:44:42
0.97954	97.0966	0.84566	1:44:44	58.838	1:44:44
0.989005	91.81339	0.814705	1:44:46	65.47	1:44:46
				57.21781	
113.7369					
				VO2/weigl	0.034601
				VCO/weigl	0.038004

VCO2	MVb/VCO2	VO2	MV/VO2	RQ		G1368	weight
0.88931	26.76569	1.1038	21.5646	0.80567			18.2
0.91859	27.42137	1.4669	17.17159	0.62984			
0.91834	26.58928	1.3154	18.56317	0.70017			
0.92453	26.70762	1.3401	18.42549	0.69362			
0.9253	25.18102	1.3106	17.77812	0.70934			
0.92666	26.90307	1.3432	18.56015	0.69422			
0.92724	38.27812	1.2961	27.38446	0.71714			
0.92597	30.1716	1.3095	21.33486	0.70752			
0.92606	21.67894	1.3883	14.46085	0.67384			
0.92899	27.66553	1.3423	19.14699	0.69446			
0.91424	28.34595	1.3749	18.84864	0.66623			
0.92799	49.0598	1.4856	30.64553	0.62616			
0.9029	20.90818	1.4564	12.9621	0.62373			
0.90888	27.88817	1.3624	18.60467	0.66844			
0.9296	28.32078	1.2666	20.78557	0.73502			
0.92323	25.54185	1.3031	18.09608	0.71039			
0.919864	28.58919	1.341575	19.6458	0.690987			
19.6185							
	MV/weigh	1.110535	28.61257				

VCO2	MVb/VCO2	VO2	MV/VO2	RQ		weight
0.88159	185.3129	1.027	159.075	0.85838		18.2
0.88435	189.4499	1.0728	156.1708	0.82519		
0.89714	171.3668	1.1447	134.3059	0.7872		
0.87447	158.0043	1.1415	121.0425	0.77687		
0.85304	157.9176	1.1087	121.5027	0.76927		
0.85107	164.4283	1.1613	120.5029	0.74428		
0.88958	196.767	1.0831	161.6102	0.82506		
0.85405	179.1464	1.123	136.2422	0.76745		
0.89573	182.8676	1.1281	145.1999	0.79511		
0.85861	180.0002	1.034	149.4681	0.83149		
0.89611	196.4491	1.1597	151.7979	0.78231		
0.87944	202.1286	1.1201	158.7001	0.78704		
0.88988	205.0838	1.1337	160.9773	0.7863		
0.92265	164.3852	1.0738	141.246	0.86617		
0.94904	138.0342	1.2107	108.2019	0.78895		
0.885117	178.0895	1.114813	141.7362	0.799405		
141.299						
	MV/weigh	6.646498	177.9675			

VCO2	MVb/VCO2	VO2	MV/VO2	RQ		G1352	weight
0.70586	49.01539	1.1861	29.16955	0.59514			18
0.71456	50.66894	1.1693	30.96382	0.61975			
0.69938	72.1582	1.2888	39.15736	0.54286			
0.70065	54.43945	1.2242	31.15749	0.57395			
0.7012	57.64974	1.1828	34.17653	0.59508			
0.70474	50.48103	1.1567	30.75646	0.61062			
0.7024	48.96782	1.2424	27.68432	0.56722			
0.70477	46.91743	1.2958	25.51783	0.54559			
0.70598	46.68546	1.2808	25.73314	0.55141			
0.70721	43.40578	1.236	24.83576	0.57292			
0.71714	43.94539	1.1728	26.87159	0.62132			
0.71139	42.02899	1.2188	24.53151	0.58445			
0.71144	44.87378	1.3374	23.87094	0.5338			
0.73106	47.3258	1.2589	27.48272	0.58098			
0.71316	45.92658	1.3948	23.48222	0.51136			
0.70833	43.44162	1.2999	23.67182	0.54756			
0.708704	49.24571	1.246594	28.06644	0.572126			
27.97578							
	MV/weight	1.948293	49.20872				

VCO2	MVb/VCO2	VO2	MV/VO2	RQ		weight
0.64353	93.40357	0.6026	99.74776	1.0801		
0.65333	84.51778	0.56331	98.02418	1.1721		18
0.6605	87.27176	0.55978	102.9744	1.2798		
0.61715	82.86478	0.7015	72.90093	0.87926		
0.68605	80.77545	0.67176	82.49375	1.025		
0.74796	74.08952	0.70563	78.53408	1.0777		
0.7298	75.52343	0.65728	83.8562	1.1124		
0.64871	84.68037	0.61188	89.77741	1.062		
0.65786	80.57793	0.59486	89.11172	1.1175		
0.70523	78.99692	0.5286	105.3935	1.3385		
0.64748	105.8272	0.5885	116.4333	1.1161		
0.68337	82.78678	0.58325	96.99786	1.1778		
0.68174	77.74812	0.65932	80.39192	1.0373		

0.67828	87.5258	0.61002	97.31976	1.124
0.70409	83.56602	0.5812	101.2354	1.2152
0.73932	88.55435	0.69036	94.83458	1.0767
0.680275	84.29436	0.619366	93.12667	1.118216
92.38132				
	MV/weigh	3.196526	84.10983	

Time	MV	Time	VCO2	MVb/VCO2	VO2	MV/VO2	RQ
0:10:50	31.409	0:10:50	0.91392	34.36734	1.2728	24.67709	0.71894
0:10:52	25.37	0:10:52	0.96356	26.32944	1.3601	18.65304	0.70843
0:10:54	43.522	0:10:54	0.9883	44.03724	1.4153	30.75108	0.70302
0:10:56	26.676	0:10:56	0.9834	27.1263	1.3942	19.13355	0.70709
0:11:00	23.104	0:11:00	0.98029	23.56854	1.3433	17.19943	0.73159
0:11:02	23.373	0:11:02	0.98439	23.74364	1.4094	16.58365	0.70001
0:11:04	28.525	0:11:04	0.99238	28.74403	1.3813	20.65084	0.7187
0:11:06	20.166	0:11:06	0.9938	20.29181	1.3556	14.87607	0.73644
0:11:08	23.164	0:11:08	0.98979	23.40294	1.4936	15.50884	0.66358
0:11:10	23.882	0:11:10	1.0415	22.93039	1.385	17.24332	0.75458
0:11:12	21.956	0:11:12	1.0414	21.08316	1.3699	16.02745	0.76119
0:11:14	20.949	0:11:14	1.0058	20.8282	1.3656	15.34051	0.74218
0:11:16	20.526	0:11:16	0.96951	21.17152	1.3903	14.76372	0.7155
0:11:18	23.102	0:11:18	0.95478	24.19615	1.4842	15.56529	0.64683
0:11:20	17.725	0:11:20	0.9329	18.99989	1.279	13.85848	0.73213
	24.8966		0.92368	25.38804	1.4151	18.05549	0.716014

VO2/weigh	0.077753	17.59353					
VCO/weigh	0.050752						
			MV/weigh	1.367945	26.95371		

Time	MV	Time	VCO2	MVb/VCO2	VO2	MV/VO2	RQ
0:25:50	136.26	0:25:50	1.0698	127.3696	1.2163	112.0283	0.88454
0:25:52	125.52	0:25:52	1.086	115.5801	1.1787	106.4902	0.93068
0:25:54	121.74	0:25:54	1.0963	111.0462	1.2129	100.371	0.90371
0:25:56	147.4	0:25:56	1.1126	132.4825	1.2186	120.9585	0.91639
0:25:58	131.64	0:25:58	1.0722	122.7756	1.2232	107.6194	0.88604
0:26:00	152.99	0:26:00	1.1173	136.9283	1.0856	140.9267	1.0341
0:26:02	124.52	0:26:02	1.0703	116.3412	1.0992	113.2824	0.97377
0:26:04	138.55	0:26:04	1.0573	131.0413	1.279	108.3268	0.82744
0:26:06	123.22	0:26:06	1.119	110.1162	1.1829	104.1677	0.95435
0:26:08	131.3	0:26:08	1.1007	119.2877	1.1593	113.258	0.95166
0:26:10	130.32	0:26:10	1.0939	119.1334	1.191	109.4207	0.91967
0:26:12	143.87	0:26:12	1.0991	130.898	1.1746	122.4842	0.95257
0:26:14	131.58	0:26:14	1.0519	125.0879	1.2115	108.6092	0.87107
0:26:16	142.93	0:26:16	1.0646	134.257	1.1627	122.9294	0.91665
0:26:18	128.91	0:26:18	1.0626	121.3156	1.1883	108.4827	0.90184
	134.05		1.084907	123.5774	1.185587	113.2903	0.921632

VO2/weigh	0.065142	113.0664
VCO/weigh	0.05961	

MV/weigh	7.365385	123.559

Time	MV	Time	VCO2	MVb/VCO2	VO2	MV/VO2	RQ
0:13:50	33.714	0:13:50	0.68385	49.30029	1.1905	28.31919	0.57588
0:13:52	32.421	0:13:52	0.68172	47.55765	1.2018	26.97703	0.57878
0:13:54	31.023	0:13:54	0.68224	45.47227	1.3042	23.787	0.52468
0:13:56	31.515	0:13:56	0.68649	45.90744	1.2476	25.2605	0.55101
0:13:58	31.18	0:13:58	0.69389	44.93508	1.3033	23.92389	0.53927
0:14:00	31.479	0:14:00	0.68557	45.91654	1.2781	24.62953	0.53773
0:14:02	29.964	0:14:02	0.68291	43.87694	1.3229	22.65024	0.51695
0:14:04	32.643	0:14:04	0.68432	47.70137	1.2932	25.24204	0.5299
0:14:06	27.658	0:14:06	0.69249	39.93993	1.3059	21.17926	0.53499
0:14:08	28.17	0:14:08	0.70013	40.23538	1.2006	23.46327	0.58423
0:14:10	29.414	0:14:10	0.69798	42.14161	1.2886	22.82632	0.54293
0:14:12	28.653	0:14:12	0.69838	41.02781	1.2869	22.26513	0.54763
0:14:14	26.439	0:14:14	0.7048	37.51277	1.2416	21.2943	0.57372
0:14:16	28.982	0:14:16	0.71873	40.3239	1.312	22.08994	0.55273
0:14:18	25.935	0:14:18	0.73101	35.47831	1.3098	19.80073	0.56078
0:14:20	25.963	0:14:20	0.71694	36.21363	1.2415	20.91261	0.57755
	29.69706		0.696341	42.72131	1.270531	23.41381	0.551798

VO2/weigh	0.070585	23.37374
VCO/weigh	0.038686	

MV/weigh	1.649837	42.64732

Time	MV	Time	VCO2	MVb/VCO2	VO2	MV/VO2	RQ
0:25:06	95.95	0:25:06	0.81776	117.3327	0.90022	106.5851	0.90919
0:25:08	96.539	0:25:08	0.77976	123.806	0.90442	106.7413	0.86433
0:25:10	100.5	0:25:10	0.81868	122.7586	0.81385	123.4871	1.0098
0:25:12	94.011	0:25:12	0.79211	118.6843	0.83954	111.9792	0.96059
0:25:14	90.386	0:25:14	0.835	108.2467	0.93858	96.30079	0.89607
0:25:16	108.06	0:25:16	0.85812	125.9264	0.9126	118.4089	0.94168
0:25:18	92.265	0:25:18	0.84216	109.5576	0.91991	100.2979	0.92036
0:25:20	84.906	0:25:20	0.86021	98.7038	0.9592	88.51751	0.90997
0:25:22	86.07	0:25:22	0.86146	99.91178	0.9765	88.14132	0.88345
0:25:24	85.45	0:25:24	0.84857	100.6988	0.96686	88.37888	0.8826
0:25:26	102.45	0:25:26	0.83514	122.674	0.89918	113.9371	0.93407
0:25:28	97.492	0:25:28	0.86914	112.1707	0.95328	102.2701	0.91689

0:25:30	106.57	0:25:30	0.84769	125.7181	0.87398	121.9364	0.97327
0:25:32	98.236	0:25:32	0.8526	115.2193	0.86176	113.9946	0.99269
	95.63464		0.837029	114.3863	0.908563	105.784	0.928211
	VO2/weigh	0.050476	105.2592				
	VCO/weigh	0.046502					
				MV/weigh	5.313036	114.2549	

	Time	MV	Time

	Time	MV	Time

VCO2	MVb/VCO2

VCO2	MVb/VCO2

Metabolism

WT 8 weeks Baseline

**G998**	**weight**	Time	MV	Time	VCO2	MVb/VCO2	VO2	Mv/VO2
	22.7	1:06:36	25.132	1:06:36	0.93535	26.86909	1.1195	22.44931
		1:06:38	21.84	1:06:38	0.93089	23.46142	1.1509	18.97645
		1:06:40	21.855	1:06:40	0.92902	23.52479	1.1486	19.02751
		1:06:42	22.563	1:06:42	0.93035	24.25216	1.3099	17.22498
		1:06:44	28.605	1:06:44	0.92933	30.78024	1.1654	24.54522
		1:06:46	24.747	1:06:46	0.94458	26.19895	1.1587	21.35756
		1:06:48	20.354	1:06:48	0.95929	21.21778	1.2857	15.83106
		1:06:50	22.255	1:06:50	0.95984	23.18616	1.1415	19.49628
		1:06:52	25.18	1:06:52	0.92886	27.1085	1.2499	20.14561
		1:06:54	23.281	1:06:54	0.92071	25.28592	1.1956	19.47223
		1:06:56	22.253	1:06:56	0.92355	24.09507	1.1952	18.61864
		1:06:58	22.793	1:06:58	0.93302	24.42927	1.1496	19.8269
		1:07:00	21.388	1:07:00	0.93631	22.84286	1.1226	19.0522
		1:07:02	22.62	1:07:02	0.91354	24.76082	1.1882	19.0372
		1:07:04	24.002	1:07:04	0.88486	27.12519	1.1651	20.60081
		1:07:06	23.628	1:07:06	0.88934	26.56802	1.1627	20.32167
			23.281		0.928053	25.10664	1.181819	19.74898
			VO2/weigh	0.052063	19.6993			
			VCO/weigh	0.040883	MV/weigh	1.025595	25.08587	

Challenge	weight	Time	MV	Time	VCO2	MVb/VCO2	VO2	Mv/VO2
	22.7	1:44:06	137.26	1:44:06	1.2805	107.1925	1.3842	99.16197
		1:44:08	158.12	1:44:08	1.2771	123.8118	1.4371	110.0271
		1:44:10	138.4	1:44:10	1.3433	103.0299	1.3922	99.411
		1:44:12	128.89	1:44:12	1.2907	99.86054	1.4013	91.97888
		1:44:14	150.21	1:44:14	1.3012	115.4396	1.3763	109.1404
		1:44:16	188.12	1:44:16	1.3665	137.6656	1.4131	133.1258
		1:44:18	149.92	1:44:18	1.3623	110.0492	1.3582	110.3814
		1:44:20	130.98	1:44:20	1.3342	98.17119	1.4568	89.90939
		1:44:22	139.86	1:44:22	1.336	104.6856	1.3866	100.8654
		1:44:24	145.76	1:44:24	1.3089	111.3607	1.3541	107.6435
		1:44:26	134.71	1:44:26	1.3289	101.3696	1.3404	100.4999
		1:44:28	128.3	1:44:28	1.3336	96.20576	1.438	89.22114
		1:44:30	132.26	1:44:30	1.3874	95.32939	1.4254	92.78799
		1:44:32	135.62	1:44:32	1.4291	94.89889	1.4664	92.485
		1:44:34	134.3	1:44:34	1.4154	94.88484	1.4943	89.87486
		1:44:36	169.89	1:44:36	1.4827	114.5815	1.4118	120.3357
			143.9125		1.348613	106.7835	1.408513	102.3031

			VO2/weigh	0.062049	102.1734			
			VCO/weigh	0.05941	MV/weigh	6.339758	106.7115	
Metabolism								
mdx/utrn 6 weeks		Baseline						
**G810**	**weight**	Time	MV	Time	VCO2	MVb/VCO2	VO2	Mv/VO2
	18.5	0:47:20	38.504	0:47:20	0.93212	41.30799	1.4637	26.30594
		0:47:22	41.033	0:47:22	0.93203	44.02541	1.4559	28.18394
		0:47:24	40.551	0:47:24	0.92187	43.98776	1.5095	26.86386
		0:47:26	31.839	0:47:26	0.90922	35.01793	1.4237	22.36356
		0:47:28	35.074	0:47:28	0.90761	38.64435	1.5588	22.50064
		0:47:30	35.603	0:47:30	0.91821	38.77435	1.5386	23.13987
		0:47:32	35.928	0:47:32	0.93972	38.23267	1.5313	23.46242
		0:47:34	35.746	0:47:34	0.97107	36.81094	1.5333	23.31312
		0:47:36	35.529	0:47:36	0.93177	38.13065	1.5685	22.65158
		0:47:38	36.618	0:47:38	0.92612	39.53915	1.4939	24.51168
		0:47:40	37.386	0:47:40	0.92536	40.40157	1.5252	24.5122
		0:47:42	35.509	0:47:42	0.89573	39.64253	1.4864	23.88926
		0:47:44	36.428	0:47:44	0.88451	41.18438	1.403	25.96436
		0:47:46	37.438	0:47:46	0.87252	42.9079	1.4556	25.71998
		0:47:48	37.316	0:47:48	0.85721	43.53192	1.4479	25.7725
		0:47:50	31.133	0:47:50	0.86852	35.84604	1.569	19.84257
			36.35219		0.912099	39.8741	1.497769	24.31234
		VO2/weigh	0.08096	24.27089				
		VCO/weigh	0.049303		MV/weigh	1.964983	39.85551	
Challenge	weight	Time	MV	Time	VCO2	MVb/VCO2	VO2	Mv/VO2
	18.5	1:47:20	76.704	1:47:20	0.68784	111.5143	0.99554	77.04763
		1:47:22	74.264	1:47:22	0.68299	108.7337	1.0843	68.49027
		1:47:24	67.854	1:47:24	0.73021	92.92395	1.0309	65.82016
		1:47:26	71.335	1:47:26	0.74611	95.60923	1.0504	67.91222
		1:47:28	67.176	1:47:28	0.6913	97.17344	1.0429	64.4127
		1:47:30	72.82	1:47:30	0.75495	96.45672	0.9829	74.08689
		1:47:32	71.048	1:47:32	0.70305	101.0568	0.98734	71.959
		1:47:34	69.941	1:47:34	0.70754	98.85095	1.0102	69.2348
		1:47:36	69.86	1:47:36	0.7251	96.34533	1.0467	66.7431
		1:47:38	72.332	1:47:38	0.7244	99.85091	0.99984	72.34357
		1:47:40	71.876	1:47:40	0.70633	101.7598	1.0403	69.09161

1:47:42	70.44	1:47:42	0.73805	95.44069	1.1149	63.18055
1:47:44	70.97	1:47:44	0.7423	95.60824	1.0725	66.17249
1:47:46	71.115	1:47:46	0.73221	97.12378	0.95596	74.39119
1:47:48	72.548	1:47:48	0.6933	104.6416	1.0367	69.97974
1:47:50	68.693	1:47:50	0.70041	98.07541	1.2329	55.7166
	71.186		0.716631	99.4478	1.042768	68.53641
VO2/weigh	0.056366	68.26642				
VCO/weigh	0.038737		MV/weigh	3.847892	99.3343	

RQ	G1168	weight	Time	MV	Time	VCO2
0.83587		26.4	0:51:10	28.136	0:51:10	0.67954
0.80986			0:51:12	28.948	0:51:12	0.66475
0.81299			0:51:14	28.393	0:51:14	0.65591
0.71196			0:51:16	29.575	0:51:16	0.64744
0.79965			0:51:18	29.329	0:51:18	0.66305
0.82295			0:51:20	29.271	0:51:20	0.66712
0.75055			0:51:22	27.245	0:51:22	0.65749
0.8428			0:51:24	29.068	0:51:24	0.65664
0.74865			0:51:26	29.894	0:51:26	0.65433
0.77143			0:51:28	27.435	0:51:28	0.66238
0.77413			0:51:30	28.379	0:51:30	0.66041
0.81291			0:51:32	29.304	0:51:32	0.66242
0.84597			0:51:34	28.186	0:51:34	0.6654
0.77003			0:51:36	27.721	0:51:36	0.66263
0.76536			0:51:38	26.509	0:51:38	0.6525
0.77334				28.49287		0.660801
0.790528						
				VO2/weigh	0.042966	25.11919
				VCO/weigh	0.02503	

RQ		weight	Time	MV	Time	VCO2
0.92588		26.4	1:51:08	154.73	1:51:08	1.17
0.89034			1:51:10	165.66	1:51:10	1.2352
0.966			1:51:12	158.82	1:51:12	1.2408
0.92222			1:51:14	159.24	1:51:14	1.2131
0.95244			1:51:16	154.37	1:51:16	1.208
0.96791			1:51:18	163.55	1:51:18	1.2525
1.0036			1:51:20	161.64	1:51:20	1.2026
0.91644			1:51:22	139.47	1:51:22	1.2567
0.968			1:51:24	153.59	1:51:24	1.2536
0.97087			1:51:26	181.35	1:51:26	1.2362
0.99547			1:51:28	160.43	1:51:28	1.2493
0.9279			1:51:30	174.39	1:51:30	1.2366
0.9744			1:51:32	162.77	1:51:32	1.1954
0.97795			1:51:34	164.35	1:51:34	1.2087
0.94948			1:51:36	157.57	1:51:36	1.1941
1.0514			1:51:38	158.14	1:51:38	1.2351
0.960019				160.6294		1.224244
				VO2/weigh	0.056687	107.3349

				VCO/weigh	0.046373	

RQ	**G1214**	**weight**	Time	MV	Time	VCO2
0.63857		21.5	0:44:12	33.139	0:44:12	0.85396
0.64099			0:44:14	34.881	0:44:14	0.855
0.61216			0:44:16	32.153	0:44:16	0.85279
0.63904			0:44:18	32.088	0:44:18	0.81927
0.58606			0:44:20	30.244	0:44:20	0.82649
0.59965			0:44:22	32.106	0:44:22	0.82758
0.61451			0:44:24	36.621	0:44:24	0.84187
0.63769			0:44:26	40.166	0:44:26	0.83233
0.59482			0:44:28	39.447	0:44:28	0.84633
0.62039			0:44:30	31.231	0:44:30	0.81344
0.60786			0:44:32	34.814	0:44:32	0.80391
0.60432			0:44:34	28.436	0:44:34	0.79798
0.6333			0:44:36	27.818	0:44:36	0.7893
0.60011			0:44:38	30.388	0:44:38	0.80886
0.59363			0:44:40	42.801	0:44:40	0.84687
0.55357			0:44:42	26.843	0:44:42	0.83894
0.611042				33.3235		0.828433
				VO2/weigh	0.062056	24.97616
				VCO/weigh	0.038532	

RQ	weight	Time	MV	Time	VCO2
0.71196	21.5	1:38:12	58.58	1:38:12	0.76508
0.63137		1:38:14	64.334	1:38:14	0.78825
0.71004		1:38:16	65.085	1:38:16	0.779
0.71174		1:38:18	65.153	1:38:18	0.75497
0.6646		1:38:20	68.044	1:38:20	0.77255
0.78161		1:38:22	63.536	1:38:22	0.78409
0.72344		1:38:24	60.166	1:38:24	0.73533
0.70819		1:38:26	54.818	1:38:26	0.73471
0.7013		1:38:28	59.474	1:38:28	0.79115
0.72598		1:38:30	68.818	1:38:30	0.81283
0.67861		1:38:32	58.128	1:38:32	0.82753

0.66965	1:38:34	56.641	1:38:34	0.79037
0.69594	1:38:36	59.411	1:38:36	0.78663
0.76848	1:38:38	70.68	1:38:38	0.83858
0.66972	1:38:40	55.47	1:38:40	0.85603
0.56809	1:38:42	59.491	1:38:42	0.82872
0.695045		61.73931		0.790364
		VO2/weigh	0.043499	66.01529
		VCO/weigh	0.036761	

MVb/VCO2	VO2	Mv/VO2	RQ	G1169	weight	Time	MV
41.40448	1.0954	25.68559	0.62453		25.2	1:36:54	23.403
43.5472	1.1122	26.02769	0.59889			1:36:56	24.395
43.28795	1.112	25.53327	0.5976			1:36:58	25.16
45.67991	1.1445	25.84098	0.56702			1:37:00	24.22
44.23347	1.0551	27.79737	0.63311			1:37:02	24.744
43.87666	1.0942	26.75105	0.61406			1:37:04	22.649
41.43789	1.322	20.60893	0.5019			1:37:06	23.437
44.26779	1.1453	25.38025	0.5742			1:37:08	22.74
45.68643	1.176	25.42007	0.56173			1:37:10	22.343
41.41882	1.0688	25.66897	0.625			1:37:12	22.719
42.97179	1.0974	25.86022	0.60432			1:37:14	24.143
44.23779	1.1698	25.05044	0.5666			1:37:16	22.311
42.35948	1.1109	25.37222	0.60009			1:37:18	19.664
41.83481	1.153	24.0425	0.57672			1:37:20	19.785
40.62682	1.158	22.89206	0.56761			1:37:22	33.243
43.12475	1.134307	25.19544	0.587559			1:37:24	35.361
							24.39481
							VO2/weigh
MV/weigh	1.079275	43.1187					VCO/weigh

MVb/VCO2	VO2	Mv/VO2	RQ		weight	Time	MV
132.2479	1.3271	116.5926	0.88163		25.2	1:51:54	149.85
134.1159	1.4111	117.3978	0.87806			1:51:56	148.65
127.9981	1.5143	104.8801	0.82279			1:51:58	145.03
131.267	1.5934	99.93724	0.76374			1:52:00	143.96
127.7897	1.5476	99.748	0.78136			1:52:02	148.86
130.5788	1.5282	107.0213	0.82058			1:52:04	142.23
134.4088	1.6058	100.6601	0.75026			1:52:06	150.66
110.9811	1.537	90.7417	0.81934			1:52:08	153.15
122.5191	1.5332	100.1761	0.81831			1:52:10	136.43
146.6996	1.5049	120.5063	0.8222			1:52:12	145.41
128.4159	1.5489	103.5767	0.80691			1:52:14	158.27
141.0238	1.4928	116.8207	0.82878			1:52:16	148.49
136.1636	1.4311	113.7377	0.8357			1:52:18	140.93
135.9725	1.4242	115.3981	0.86204			1:52:20	143.01
131.9571	1.5042	104.7534	0.80001			1:52:22	154.89
128.0382	1.4406	109.7737	0.86356				147.3213
131.2611	1.496525	107.6076	0.822204				VO2/weigh
							VCO/weigh

MV/weigh	6.084446	131.207

						Time	MV
MVb/VCO2	VO2	Mv/VO2	RQ	**G1215**	**weight**	1:14:04	30.769
38.80627	1.4305	23.16603	0.59721		16.7	1:14:06	29.521
40.79649	1.4473	24.10074	0.59414			1:14:08	30.957
37.7033	1.3711	23.45051	0.62521			1:14:10	30.385
39.16658	1.3039	24.60925	0.62865			1:14:12	30.66
36.5933	1.3138	23.02025	0.64075			1:14:14	33.232
38.79504	1.3757	23.33794	0.60603			1:14:16	27.237
43.49959	1.2672	28.89915	0.66611			1:14:18	28.613
48.2573	1.3738	29.23715	0.6083			1:14:20	27.169
46.60948	1.3817	28.54961	0.61646			1:14:22	25.263
38.39374	1.2245	25.5051	0.6672			1:14:24	26.47
43.30584	1.2551	27.73803	0.64662			1:14:26	32.521
35.63498	1.4156	20.0876	0.56407			1:14:28	24.407
35.24389	1.2506	22.24372	0.63473			1:14:30	26.494
37.56892	1.369	22.19722	0.59154			1:14:32	27.83
50.54022	1.3131	32.59538	0.64523			1:14:34	29.204
31.99633	1.2545	21.39737	0.66957				28.79575
40.18195	1.334213	25.00844	0.625114				VO2/weigh
							VCO/weigh

MV/weigh	1.54993	40.22476

					Time	MV
MVb/VCO2	VO2	Mv/VO2	RQ	weight	1:59:04	40.232
76.56716	0.9192	63.72933	0.83233	16.7	1:59:06	39.229
81.61624	0.91965	69.95487	0.8613		1:59:08	39.955
83.54942	0.93228	69.81272	0.84063		1:59:10	41.042
86.29879	0.95968	67.89034	0.78837		1:59:12	42.684
88.07715	0.89538	75.99455	0.86286		1:59:14	45.132
81.03151	0.89131	71.28384	0.89665		1:59:16	47.176
81.82177	0.85279	70.55195	0.87882		1:59:18	46.339
74.61175	0.94628	57.93	0.78297		1:59:20	41.891
75.17411	0.90921	65.41283	0.86973		1:59:22	41.535
84.66469	1.016	67.73425	0.81133		1:59:24	36.926
70.24277	1.0235	56.79336	0.81267		1:59:26	41.435

71.6639	0.85146	66.52221	0.93254	1:59:28	45.919
75.52598	0.9722	61.10985	0.81766	1:59:30	44.046
84.28534	0.86819	81.41075	0.9685	1:59:32	38.046
64.79913	0.93581	59.27485	0.92218	1:59:34	38.568
71.78661	1.0707	55.56272	0.78081		41.88469
78.23227	0.935228	66.31053	0.853709		VO2/weigh
					VCO/weigh
MV/weigh	2.871596	78.11506			

Time	VCO2	MVb/VCO2	VO2	Mv/VO2	RQ		G1368
1:36:54	0.67128	34.86325	1.0407	22.48775	0.65405		
1:36:56	0.66418	36.7295	1.0207	23.90026	0.65931		
1:36:58	0.65966	38.14086	1.0363	24.27868	0.64082		
1:37:00	0.64741	37.41061	0.99469	24.34929	0.65129		
1:37:02	0.65084	38.01856	0.96758	25.57308	0.67725		
1:37:04	0.6446	35.13652	0.94058	24.07982	0.69826		
1:37:06	0.64648	36.25325	1.0398	22.53991	0.62528		
1:37:08	0.63944	35.56237	1.0818	21.02052	0.59476		
1:37:10	0.63485	35.19414	0.99901	22.36514	0.63695		
1:37:12	0.64086	35.4508	1.0419	21.80536	0.61552		
1:37:14	0.64202	37.60475	1.0556	22.87135	0.60941		
1:37:16	0.63383	35.20029	1.0277	21.70964	0.62053		
1:37:18	0.63825	30.80924	1.082	18.17375	0.5924		
1:37:20	0.62903	31.45319	1.0289	19.22927	0.61271		
1:37:22	0.6336	52.46686	0.97335	34.15318	0.66369		
1:37:24	0.64036	55.2205	0.9567	36.96143	0.66934		
	0.644793	37.84467	1.017957	24.09365	0.638848		
0.040395	23.96449						
0.025587		MV/weigh	0.968048	37.83355			

Time	VCO2	MVb/VCO2	VO2	Mv/VO2	RQ
1:48:08	1.1452	130.8505	1.4039	106.7384	0.81647
1:48:10	1.1284	131.7352	1.4921	99.62469	0.75741
1:48:12	1.168	124.1695	1.4152	102.4802	0.82844
1:48:14	1.1723	122.8013	1.3638	105.558	0.86437
1:48:16	1.1578	128.5714	1.4552	102.2952	0.79805
1:48:18	1.1275	126.1463	1.4484	98.19801	0.77846
1:48:20	1.1131	135.3517	1.4987	100.5271	0.74379
1:48:22	1.169	131.0094	1.444	106.0596	0.81108
1:48:24	1.1406	119.6125	1.4968	91.14778	0.76342
1:48:26	1.1815	123.0724	1.5117	96.18972	0.7846
1:48:28	1.1444	138.2995	1.4677	107.8354	0.77982
1:48:30	1.1585	128.1744	1.4434	102.8752	0.80548
1:48:32	1.1362	124.0363	1.4843	94.94711	0.77014
1:48:34	1.1754	121.6692	1.3555	105.5035	0.86989
1:48:36	1.152	134.4531	1.3687	113.1658	0.84458
	1.151327	127.9969	1.443293	102.2097	0.801067
0.057274	102.073				
0.045688					

		MV/weigh	5.846085	127.9579

Time	VCO2	MVb/VCO2	VO2	Mv/VO2	RQ	G1352
1:14:04	0.6457	47.65216	1.1821	26.0291	0.55259	
1:14:06	0.6341	46.55575	1.0717	27.54596	0.59723	
1:14:08	0.6318	48.9981	1.0869	28.48192	0.58201	
1:14:10	0.63946	47.51665	1.0216	29.74256	0.62793	
1:14:12	0.61759	49.64459	1.0666	28.74555	0.58191	
1:14:14	0.60561	54.8736	1.0069	33.00427	0.60302	
1:14:16	0.59222	45.99135	1.0136	26.87155	0.58769	
1:14:18	0.58713	48.73367	1.0148	28.1957	0.58132	
1:14:20	0.60353	45.01682	0.98489	27.58582	0.61549	
1:14:22	0.60274	41.91359	1.0376	24.34753	0.586	
1:14:24	0.60884	43.47612	1.1059	23.93526	0.55226	
1:14:26	0.61323	53.0323	1.0356	31.40305	0.59667	
1:14:28	0.614	39.75081	0.9338	26.13729	0.66294	
1:14:30	0.61544	43.04888	0.86352	30.6814	0.71786	
1:14:32	0.6118	45.48872	1.0168	27.37018	0.60518	
1:14:34	0.61181	47.73377	1.0241	28.51675	0.59741	
	0.614688	46.83918	1.029151	28.03712	0.602969	
0.061626	27.98011					
0.036808		MV/weigh	1.724296	46.84616		

Time	VCO2	MVb/VCO2	VO2	Mv/VO2	RQ
1:59:04	0.51231	78.53058	0.61712	65.19316	0.85802
1:59:06	0.52085	75.31727	0.54565	71.89407	0.9837
1:59:08	0.53019	75.35978	0.59204	67.48699	0.91399
1:59:10	0.512	80.16016	0.47162	87.02345	1.0973
1:59:12	0.51927	82.20001	0.55907	76.34822	0.93024
1:59:14	0.56693	79.60771	0.4796	94.10342	1.2694
1:59:16	0.54196	87.04701	0.55753	84.61607	0.98712
1:59:18	0.55865	82.94818	0.55902	82.89328	1.0217
1:59:20	0.56437	74.22613	0.56956	73.54976	1.0055
1:59:22	0.53282	77.95315	0.65037	63.86365	0.82014
1:59:24	0.55092	67.02607	0.6493	56.87048	0.87886
1:59:26	0.56966	72.73637	0.57311	72.29851	1.0026

1:59:28	0.52022	88.26842	0.51833	88.59028	1.042
1:59:30	0.55586	79.23938	0.52822	83.38571	1.0568
1:59:32	0.52891	71.93284	0.4811	79.08127	1.1485
1:59:34	0.5294	72.85229	0.52882	72.93219	1.0011
	0.538395	77.83783	0.555029	76.25816	1.001061
0.033235	75.464				
0.032239		MV/weigh	2.508065	77.79546	

**weight**	Time	MV	Time	VCO2	MVb/VCO2	VO2	MV/VO2	RQ
20.8	0:56:16	24.012	0:56:16	0.84895	28.28435	1.3709	17.5155	0.61928
	0:56:18	20.746	0:56:18	0.84751	24.47877	1.2548	16.53331	0.67669
	0:56:20	22.429	0:56:20	0.8799	25.4904	1.3206	16.98395	0.67416
	0:56:22	24.583	0:56:22	0.85584	28.72383	1.2393	19.8362	0.69407
	0:56:24	20.332	0:56:24	0.83242	24.42517	1.2583	16.15831	0.66383
	0:56:26	32.631	0:56:26	0.84059	38.81916	1.1835	27.57161	0.71491
	0:56:28	24.895	0:56:28	0.836	29.77871	1.1468	21.70823	0.73348
	0:56:30	24.813	0:56:30	0.83001	29.89482	1.3058	19.00214	0.64648
	0:56:32	21.344	0:56:32	0.8351	25.55862	1.2921	16.51885	0.65047
	0:56:34	22.714	0:56:34	0.83003	27.36528	1.2168	18.667	0.68417
	0:56:36	23.035	0:56:36	0.82412	27.95103	1.3032	17.67572	0.63458
	0:56:38	19.958	0:56:38	0.82207	24.27774	1.1863	16.82374	0.6956
	0:56:40	20.708	0:56:40	0.82253	25.17598	1.2335	16.788	0.66865
	0:56:42	22.794	0:56:42	0.8344	27.31783	1.276	17.86364	0.65753
	0:56:44	20.815	0:56:44	0.83026	25.07046	1.2469	16.6934	0.67454
	0:56:46	20.49	0:56:46	0.82474	24.84419	1.2204	16.78958	0.67723
		22.89369		0.837154	27.34102	1.25345	18.32057	0.672854
			VO2/weigh	0.060262	18.26454			
			VCO/weigh	0.040248				
						MV/weigh	1.100658	27.34703

**weight**	Time	MV	Time	VCO2	MVb/VCO2	VO2	MV/VO2	RQ
20.8	1:17:16	142.74	1:17:16	1.1823	120.7308	1.476	96.70732	0.80098
	1:17:18	150.33	1:17:18	1.3013	115.5229	1.3712	109.6339	0.94992
	1:17:20	155.63	1:17:20	1.2925	120.4101	1.3792	112.8408	0.94711
	1:17:22	171.72	1:17:22	1.2279	139.8485	1.4184	121.066	0.86658
	1:17:24	169.97	1:17:24	1.2725	133.5717	1.419	119.7815	0.89952
	1:17:26	168.43	1:17:26	1.2485	134.9059	1.3273	126.8967	0.94598
	1:17:28	180.19	1:17:28	1.2623	142.7474	1.2853	140.193	0.98599
	1:17:30	167.41	1:17:30	1.2412	134.8775	1.3535	123.6867	0.91845
	1:17:32	145.3	1:17:32	1.263	115.0435	1.3865	104.7962	0.91202
	1:17:34	162.37	1:17:34	1.2306	131.9438	1.3576	119.6008	0.91707
	1:17:36	185.36	1:17:36	1.2818	144.6091	1.3585	136.4446	0.94413
	1:17:38	168.87	1:17:38	1.2059	140.0365	1.3536	124.7562	0.89303
	1:17:40	161.7	1:17:40	1.2281	131.6668	1.3805	117.1315	0.90501
	1:17:42	161.72	1:17:42	1.2584	128.5124	1.4491	111.6003	0.87272
	1:17:44	175.78	1:17:44	1.2971	135.5177	1.3896	126.4968	0.93569
		164.5013		1.252893	131.3296	1.380353	119.4422	0.912947
			VO2/weigh	0.066363	119.1734			
			VCO/weigh	0.060235				
						MV/weigh	7.908718	131.2972

weight	Time	MV	Time	VCO2	MVb/VCO2	VO2	MV/VO2	RQ
20.2	0:49:32	29.422	0:49:32	1.2835	22.92326	1.7636	16.68292	0.73257
	0:49:34	25.648	0:49:34	1.2642	20.28793	1.7332	14.79806	0.73075
	0:49:36	25.776	0:49:36	1.2487	20.64227	1.6466	15.65408	0.76122
	0:49:38	26.046	0:49:38	1.2398	21.00823	1.6359	15.92151	0.76228
	0:49:40	25.691	0:49:40	1.2251	20.97053	1.6641	15.43838	0.73774
	0:49:42	27.784	0:49:42	1.2213	22.74953	1.5662	17.73975	0.7832
	0:49:44	25.025	0:49:44	1.2056	20.7573	1.6292	15.3603	0.74131
	0:49:46	24.633	0:49:46	1.2017	20.49846	1.6019	15.37736	0.75042
	0:49:48	25.912	0:49:48	1.1844	21.87774	1.6971	15.2684	0.6999
	0:49:50	25.804	0:49:50	1.1683	22.08679	1.809	14.26423	0.6471
	0:49:52	27.022	0:49:52	1.1466	23.56707	1.6489	16.38789	0.69754
	0:49:54	32.203	0:49:54	1.1317	28.45542	1.6445	19.58224	0.6903
	0:49:56	26.931	0:49:56	1.0896	24.71641	1.6606	16.21763	0.66192
	0:49:58	27.275	0:49:58	1.0246	26.62014	1.4362	18.99109	0.71436
	0:50:00	27.624	0:50:00	1.0105	27.33696	1.3835	19.96675	0.73065
	0:50:02	35.815	0:50:02	0.99128	36.13005	1.3558	26.41614	0.73112
		27.41319		1.164805	23.78926	1.617269	17.12917	0.723274
			VO2/weigh	0.080063	16.9503			
			VCO/weigh	0.057664				
						MV/weigh	1.357088	23.53457

weight	Time	MV	Time	VCO2	MVb/VCO2	VO2	MV/VO2	RQ
20.2	1:16:32	82.883	1:16:32	0.65088	127.3399	0.66401	124.8219	0.9805
	1:16:34	81.881	1:16:34	0.71148	115.0855	0.71282	114.8691	1.0035
	1:16:36	80.27	1:16:36	0.74782	107.3387	0.70541	113.792	1.0631
	1:16:38	80.577	1:16:38	0.76221	105.715	0.80016	100.7011	0.95654
	1:16:40	79.077	1:16:40	0.86771	91.13298	0.67907	116.449	1.3056
	1:16:42	89.335	1:16:42	0.8077	110.6042	0.75169	118.8455	1.1072
	1:16:44	84.781	1:16:44	0.78073	108.592	0.72629	116.7316	1.0768
	1:16:46	91.549	1:16:46	0.85469	107.1137	0.80383	113.891	1.0883
	1:16:48	84.457	1:16:48	0.80073	105.475	0.7493	112.7145	1.0739
	1:16:50	82.956	1:16:50	0.81438	101.864	0.82559	100.4809	1.0104
	1:16:52	86.658	1:16:52	0.76978	112.575	0.78124	110.9237	1.0112
	1:16:54	90.233	1:16:54	0.78958	114.2797	0.76745	117.5751	1.032

1:16:56	81.998	1:16:56	0.73984	110.8321	0.69518	117.9522		1.0824
1:16:58	86.792	1:16:58	0.75633	114.7541	0.77203	112.4205		1.0324
1:17:00	79.564	1:17:00	0.71309	111.5764	0.86575	91.90182		0.82478
	84.20073		0.77113	109.6185	0.753321	112.2713		1.043241
			VO2/weigh	0.037293	111.7727			
			VCO/weigh	0.038175				
						MV/weigh		4.168353

Time	MV	Time

Time	MV	Time

109.1914

VCO2	MVb/VCO2

VCO2	MVb/VCO2

Metabolism
WT 10 weeks

G1168	weight	Time	MV	Time	VCO2	MVb/VCO2	VO2
	24.9	1:34:26	45.651	1:34:26	0.8495	53.73867	1.2891
		1:34:28	44.988	1:34:28	0.85177	52.81708	1.2982
		1:34:30	44.328	1:34:30	0.85245	52.0007	1.4074
		1:34:32	46.557	1:34:32	0.8558	54.40173	1.4231
		1:34:34	49.882	1:34:34	0.84856	58.78429	1.3607
		1:34:36	45.337	1:34:36	0.85429	53.0698	1.3477
		1:34:38	46.021	1:34:38	0.84577	54.41314	1.2683
		1:34:40	45.568	1:34:40	0.84586	53.8718	1.3033
		1:34:42	45.662	1:34:42	0.85618	53.33224	1.2852
		1:34:44	44.509	1:34:44	0.8552	52.04514	1.2235
		1:34:46	44.775	1:34:46	0.85669	52.26511	1.3591
		1:34:48	44.83	1:34:48	0.85337	52.5329	1.2703
		1:34:50	45.828	1:34:50	0.83864	54.64562	1.3419
		1:34:52	44.034	1:34:52	0.83087	52.99746	1.3486
		1:34:54	43.934	1:34:54	0.84586	51.94004	1.404
			45.46027		0.849387	53.52371	1.328693
			VO2/weigh	0.053361	34.21427		
			VCO/weigh	0.034112		MV/weigh	1.825714

Challenge	weight	Time	MV	Time	VCO2	MVb/VCO2	VO2
	24.9	1:46:26	187.02	1:46:26	1.1437	163.5219	1.1535
		1:46:28	180.35	1:46:28	1.289	139.9147	1.2736
		1:46:30	168.38	1:46:30	1.2945	130.0734	1.4207
		1:46:32	178.73	1:46:32	1.3235	135.0434	1.382
		1:46:34	190.1	1:46:34	1.3811	137.6439	1.4841
		1:46:36	184.1	1:46:36	1.386	132.8283	1.5218
		1:46:38	177.28	1:46:38	1.3573	130.6122	1.5289
		1:46:40	194.97	1:46:40	1.3738	141.9202	1.6549
		1:46:42	183.44	1:46:42	1.4033	130.7204	1.5463
		1:46:44	180.53	1:46:44	1.419	127.2234	1.6216
		1:46:46	178.63	1:46:46	1.4331	124.6459	1.6361
		1:46:48	187.16	1:46:48	1.4383	130.1258	1.5727
		1:46:50	179.47	1:46:50	1.4322	125.3107	1.6528
		1:46:52	178.68	1:46:52	1.4704	121.518	1.6564
		1:46:54	181.78	1:46:54	1.4099	128.9311	1.7189
			182.0413		1.37034	133.3356	1.52162
			VO2/weigh	0.061109	119.6365		
			VCO/weigh	0.055034			

								MV/weigh	7.310897

Metabolism									
mdx/utrn 10 weeks				Time	MV	Time	VCO2	MVb/VCO2	VO2
	**G1214**	**weight**		0:44:10	34.682	0:44:10	0.78938	43.93575	1.2088
			21	0:44:12	29.58	0:44:12	0.78593	37.63694	1.1219
				0:44:14	51.188	0:44:14	0.78686	65.0535	1.1799
				0:44:16	31.307	0:44:16	0.77585	40.35187	1.1143
				0:44:18	28.342	0:44:18	0.78048	36.31355	1.1817
				0:44:20	30.034	0:44:20	0.79672	37.69706	1.1621
				0:44:22	33.56	0:44:22	0.79674	42.12165	1.2234
				0:44:24	31.989	0:44:24	0.78023	40.99945	1.1245
				0:44:26	32.559	0:44:26	0.76337	42.65166	1.023
				0:44:28	35.661	0:44:28	0.77333	46.11356	1.0833
				0:44:30	36.092	0:44:30	0.76767	47.01499	0.99732
				0:44:32	33.864	0:44:32	0.73986	45.77082	1.0768
				0:44:34	32.946	0:44:34	0.72258	45.59495	1.0903
				0:44:36	36.288	0:44:36	0.71877	50.48625	0.99037
				0:44:38	39.356	0:44:38	0.71145	55.31801	1.0519
				0:44:40	33.549	0:44:40	0.68361	49.07623	0.85487
					34.43731		0.760802	45.38352	1.092779
					VO2/weigh	0.052037	31.51353		
					VCO/weigh	0.036229			
								MV/weigh	1.639872

			Time	MV	Time	VCO2	MVb/VCO2	VO2
Challenge	weight		1:47:10	67.519	1:47:10	0.78755	85.73297	0.7875
		21	1:47:12	79.304	1:47:12	0.78845	100.5822	0.78707
			1:47:14	77.491	1:47:14	0.79247	97.78414	0.83003
			1:47:16	61.803	1:47:16	0.80924	76.37166	0.85486
			1:47:18	57.687	1:47:18	0.84121	68.57622	0.78021
			1:47:20	63.398	1:47:20	0.79524	79.72184	0.81118
			1:47:22	57.985	1:47:22	0.85308	67.97135	0.78178
			1:47:24	71.583	1:47:24	0.86182	83.06027	0.78705
			1:47:26	49.715	1:47:26	0.83347	59.64822	0.88276
			1:47:28	67.942	1:47:28	0.8197	82.88642	0.83158
			1:47:30	69.71	1:47:30	0.78959	88.28633	0.80996
			1:47:32	67.454	1:47:32	0.80026	84.29011	0.82412

1:47:34	56.338	1:47:34	0.8362	67.37383	0.86876
1:47:36	72.599	1:47:36	0.82052	88.47926	0.83991
1:47:38	61.355	1:47:38	0.86074	71.28169	0.81778
1:47:40	72.789	1:47:40	0.88955	81.82677	0.83
	65.917		0.823693	80.24208	0.820284
	VO2/weigl	0.039061	80.35872		
	VCO/weigl	0.039223		MV/weigh	3.138905

Mv/VO2	RQ	**G1169**	**weight**	Time	MV	Time	VCO2
35.41308	0.66734		25.9	1:26:10	22.111	1:26:10	0.87725
34.65414	0.66045			1:26:12	22.321	1:26:12	0.86509
31.49638	0.61413			1:26:14	24.34	1:26:14	0.86286
32.7152	0.60279			1:26:16	22.514	1:26:16	0.85574
36.65907	0.62622			1:26:18	22.354	1:26:18	0.85946
33.64028	0.63842			1:26:20	22.497	1:26:20	0.85872
36.28558	0.67024			1:26:22	22.982	1:26:22	0.85585
34.96355	0.64932			1:26:24	22.339	1:26:24	0.86014
35.5291	0.66986			1:26:26	21.456	1:26:26	0.85105
36.37842	0.69978			1:26:28	23.628	1:26:28	0.83915
32.9446	0.63055			1:26:30	21.766	1:26:30	0.84383
35.29088	0.6778			1:26:32	24.503	1:26:32	0.83532
34.15158	0.6272			1:26:34	22.862	1:26:34	0.81796
32.65164	0.62361			1:26:36	22.622	1:26:36	0.81183
31.29202	0.6071			1:26:38	32.134	1:26:38	0.81652
34.27103	0.644321			1:26:40	32.306	1:26:40	0.81146
					23.92094		0.845139
					VO2/weigh	0.048544	19.02573
53.52124					VCO/weigh	0.032631	

Mv/VO2	RQ		weight	Time	MV	Time	VCO2
162.1326	0.99212		25.9	2:11:12	185.77	2:11:12	1.1793
141.6065	1.0141			2:11:14	185.08	2:11:14	1.1867
118.519	0.91562			2:11:16	182.51	2:11:16	1.2111
129.3271	0.96239			2:11:18	194.15	2:11:18	1.2366
128.0911	0.93147			2:11:20	198.07	2:11:20	1.2393
120.9752	0.92001			2:11:22	159.52	2:11:22	1.2697
115.9526	0.89456			2:11:24	213.48	2:11:24	1.227
117.8138	0.83076			2:11:26	177.85	2:11:26	1.2999
118.6316	0.90914			2:11:28	202.46	2:11:28	1.3036
111.3283	0.87508			2:11:30	172.83	2:11:30	1.3155
109.1804	0.87667			2:11:32	181.88	2:11:32	1.3105
119.0055	0.91734			2:11:34	175.6	2:11:34	1.3215
108.5854	0.86685			2:11:36	189.11	2:11:36	1.3308
107.8725	0.8886			2:11:38	197.43	2:11:38	1.3295
105.7537	0.82158			2:11:40	190.69	2:11:40	1.3433
120.985	0.907753				187.0953		1.27362
					VO2/weigh	0.070804	102.0245
					VCO/weigh	0.049175	

132.8439

Mv/VO2	RQ			Time	MV	Time	VCO2
28.69126	0.65931	**G1215**	**weight**	0:34:26	35.61	0:34:26	0.65977
26.36599	0.703		14.3	0:34:28	32.773	0:34:28	0.65612
43.38334	0.67576			0:34:30	35.957	0:34:30	0.65494
28.09567	0.69644			0:34:32	33.122	0:34:32	0.65876
23.98409	0.66762			0:34:34	34.777	0:34:34	0.67448
25.84459	0.68648			0:34:36	32.111	0:34:36	0.65957
27.43175	0.65265			0:34:38	36.743	0:34:38	0.66542
28.44731	0.69784			0:34:40	32.847	0:34:40	0.68708
31.82698	0.74923			0:34:42	37.446	0:34:42	0.66816
32.91886	0.72261			0:34:44	34.042	0:34:44	0.66554
36.18899	0.77611			0:34:46	35.283	0:34:46	0.6823
31.44874	0.68774			0:34:48	35.73	0:34:48	0.65622
30.21737	0.67			0:34:50	36.569	0:34:50	0.66373
36.64085	0.73128			0:34:52	38.931	0:34:52	0.66258
37.4142	0.67927			0:34:54	36.592	0:34:54	0.67095
39.24456	0.80057			0:34:56	36.014	0:34:56	0.70318
31.75903	0.703494				35.28419		0.66805
					VO2/weigh	0.07777	31.72714
					VCO/weigh	0.046717	

45.26449

Mv/VO2	RQ		Time	MV	Time	VCO2
85.73841	1.0086	weight	2:10:26	37.332	2:10:26	-0.03551
100.7585	1.0063	14.3	2:10:28	37.38	2:10:28	0.053621
93.35928	0.95639		2:10:30	39.35	2:10:30	0.12382
72.29605	0.96213		2:10:32	38.886	2:10:32	0.24565
73.93779	1.0937		2:10:34	39.288	2:10:34	0.24533
78.15528	0.98771		2:10:36	41.044	2:10:36	0.2815
74.17048	1.0944		2:10:38	42.804	2:10:38	0.30219
90.95102	1.1054		2:10:40	40.408	2:10:40	0.31556
56.31769	0.94555		2:10:42	31.177	2:10:42	0.37228
81.7023	0.98787		2:10:44	34.914	2:10:44	0.33771
86.06598	0.97617		2:10:46	47.162	2:10:46	0.40831
81.84973	0.98282		2:10:48	35.688	2:10:48	0.39178

64.84875	0.9709	2:10:50	44.511	2:10:50	0.44836
86.43664	0.98291	2:10:52	38.368	2:10:52	0.48821
75.02629	1.0566	2:10:54	37.297	2:10:54	0.52479
87.69759	1.0718	2:10:56	28.717	2:10:56	0.54076
80.58199	1.011828		38.39538		0.315273
			VO2/weigh	0.030668	87.54953
80.02616			VCO/weigh	0.022047	

MVb/VCO2	VO2	Mv/VO2	RQ	G1368	weight	Time
25.2049	1.3781	16.04455	0.63656		21.2	1:40:50
25.80194	1.3506	16.52673	0.65529			1:40:52
28.20852	1.292	18.83901	0.67437			1:40:58
26.30939	1.325	16.9917	0.64691			1:41:00
26.00935	1.3363	16.72828	0.64413			1:41:02
26.1983	1.2012	18.72877	0.72343			1:41:04
26.85284	1.3501	17.02244	0.63568			1:41:06
25.97135	1.2629	17.68865	0.68359			1:41:08
25.21121	1.2077	17.766	0.71208			1:41:10
28.15706	1.1635	20.30769	0.72143			1:41:12
25.7943	1.221	17.82637	0.69513			1:41:14
29.33367	1.3283	18.44689	0.63001			1:41:16
27.95002	1.2299	18.5885	0.66533			1:41:18
27.86544	1.1404	19.8369	0.72331			1:41:20
39.35482	1.1107	28.9313	0.75048			
39.81219	1.219	26.50205	0.67063			
28.37721	1.257294	19.17349	0.679273			

MV/weigh	0.923588	28.30413

MVb/VCO2	VO2	Mv/VO2	RQ	weight	Time
				21.2	1:58:50
157.5257	1.6104	115.3564	0.73354		1:58:52
155.9619	1.7439	106.1299	0.68063		1:58:54
150.6977	1.7181	106.2278	0.71189		1:58:56
157.0031	1.8477	105.0766	0.6713		1:58:58
159.8241	1.7883	110.7588	0.6955		1:59:00
125.636	1.8203	87.63391	0.69786		1:59:02
173.9853	1.8755	113.8256	0.65471		1:59:04
136.8182	1.799	98.86048	0.72313		1:59:06
155.3084	1.9462	104.0284	0.671		1:59:08
131.3797	1.8479	93.52779	0.71297		1:59:10
138.7867	1.9358	93.95599	0.68012		1:59:12
132.8793	1.869	93.95399	0.70882		1:59:14
142.1025	1.8786	100.6654	0.71032		1:59:16
148.4994	1.9536	101.0596	0.68217		1:59:18
141.9564	1.8731	101.8045	0.71877		
147.2243	1.833827	102.191	0.696849		

MV/weigh	7.223758	146.9004

MVb/VCO2	VO2	Mv/VO2	RQ	G1352	weight	Time
53.97335	1.0824	32.89911	0.61259		20.8	0:52:06
49.9497	1.105	29.65882	0.59377			0:52:08
54.90121	0.99911	35.98903	0.6583			0:52:10
50.27931	1.1018	30.06172	0.60177			0:52:12
51.5612	1.1734	29.6378	0.57731			0:52:14
48.68475	1.2247	26.21948	0.53922			0:52:16
55.21776	1.128	32.57358	0.59146			0:52:18
47.80666	1.1868	27.67695	0.58219			0:52:20
56.04346	1.1478	32.62415	0.58461			0:52:22
51.14944	1.1208	30.37295	0.59725			0:52:24
51.71186	0.97366	36.2375	0.70971			0:52:26
54.4482	0.99645	35.85729	0.66441			0:52:28
55.0962	1.1025	33.16916	0.60441			0:52:30
58.75668	1.1235	34.65154	0.59021			0:52:32
54.5376	1.1195	32.68602	0.60179			0:52:34
51.2159	1.2084	29.80305	0.58192			0:52:36
52.83333	1.112114	31.88238	0.605683			

MV/weigh	2.467426	52.81669

MVb/VCO2	VO2	Mv/VO2	RQ	weight	Time
-1051.43	0.083307	448.1256	-0.50908	20.8	1:58:08
697.1149	0.12724	293.7755	0.30473		1:58:10
317.8	0.26692	147.4224	0.74621		1:58:12
158.2984	0.24202	160.6727	1.0558		1:58:14
160.1435	0.32122	122.3087	0.78556		1:58:16
145.8046	0.43741	93.83416	0.65703		1:58:18
141.646	0.39727	107.7454	0.76931		1:58:20
128.0517	0.47323	85.38766	0.70784		1:58:22
83.74611	0.50693	61.50159	0.78442		1:58:24
103.3846	0.52069	67.05333	0.68182		1:58:26
115.5054	0.49046	96.15871	0.83971		1:58:28
91.09194	0.54679	65.2682	0.72568		1:58:30

99.27514	0.59054	75.37339	0.75942	1:58:32
78.58913	0.656	58.4878	0.76781	1:58:34
71.07033	0.72565	51.39806	0.73612	
53.10489	0.63122	45.49444	0.86122	
87.07492	0.438556	123.7505	0.6671	
MV/weigh	2.684991	121.7846		

MV	Time	VCO2	MVb/VCO2	VO2	MV/VO2	RQ
25.969	1:40:50	0.48932	53.07161	1.0551	24.61283	0.46378
38.996	1:40:52	0.50759	76.82578	0.81898	47.61533	0.6398
28.816	1:40:58	0.48672	59.20447	0.67929	42.42076	0.73158
20.513	1:41:00	0.4796	42.77106	0.75742	27.08273	0.64981
19.124	1:41:02	0.49684	38.49126	0.84098	22.74014	0.59344
25.2	1:41:04	0.4946	50.95026	0.8715	28.91566	0.57257
19.974	1:41:06	0.5004	39.91607	0.75143	26.58132	0.67944
20.718	1:41:08	0.48547	42.67617	0.85836	24.13673	0.56849
20.395	1:41:10	0.49181	41.46927	0.78333	26.03628	0.6334
18.726	1:41:12	0.50242	37.27161	0.79646	23.51154	0.63818
18.356	1:41:14	0.50449	36.38526	0.85412	21.49113	0.61371
18.618	1:41:16	0.50334	36.98891	0.91356	20.37961	0.55583
17.565	1:41:18	0.50364	34.8761	0.79607	22.06464	0.63806
19.34	1:41:20	0.5093	37.97369	0.82947	23.31609	0.6252
22.30786		0.496824	44.91939	0.829005	27.20748	0.614521
	VO2/weigh	0.039104	26.90919			
	VCO/weigh	0.023435				
				MV/weigh	1.052257	44.9009

MV	Time	VCO2	MVb/VCO2	VO2	MV/VO2	RQ
182.29	1:58:50	0.61355	297.107	0.46286	393.834	1.3256
198.64	1:58:52	0.57188	347.3456	0.57781	343.7808	1.004
160.78	1:58:54	0.67466	238.3126	0.60097	267.5342	1.1248
172.39	1:58:56	0.68577	251.3817	0.52178	330.3883	1.4347
162.91	1:58:58	0.67496	241.3625	0.51845	314.2251	1.3047
159.46	1:59:00	0.70636	225.7489	0.54896	290.4765	1.3102
161.07	1:59:02	0.70981	226.9199	0.55904	288.1189	1.2954
192.24	1:59:04	0.68962	278.7622	0.69234	277.667	1.0085
182.85	1:59:06	0.71186	256.8623	0.62906	290.6718	1.1484
150.35	1:59:08	0.70708	212.6351	0.68208	220.4287	1.0385
163.51	1:59:10	0.70081	233.3157	0.57164	286.0367	1.2924
162.66	1:59:12	0.73749	220.5589	0.70362	231.1759	1.0931
146.91	1:59:14	0.71966	204.1381	0.749	196.1415	0.96967
166.42	1:59:16	0.71701	232.1028	0.56725	293.3803	1.3117
193.65	1:59:18	0.70027	276.5362	0.62437	310.1526	1.1259
170.4087		0.688053	249.5393	0.600615	288.9342	1.185838
	VO2/weigh	0.028331	283.7235			
	VCO/weigh	0.032455				
				MV/weigh	8.038145	247.6681

MV	Time	VCO2	MVb/VCO2	VO2	MV/VO2	RQ
30.364	0:52:06	0.48924	62.06361	0.83776	36.24427	0.58399
31.762	0:52:08	0.48395	65.63075	0.88405	35.92783	0.54878
34.529	0:52:10	0.48804	70.75035	0.8206	42.07775	0.60165
34.512	0:52:12	0.48929	70.53486	0.92848	37.17043	0.53308
31.151	0:52:14	0.48962	63.62281	0.85339	36.50265	0.57846
30.304	0:52:16	0.49001	61.84364	0.85161	35.58436	0.58213
33.268	0:52:18	0.49078	67.78597	0.86815	38.32057	0.5675
31.615	0:52:20	0.49824	63.45336	0.95645	33.05452	0.52234
28.202	0:52:22	0.48964	57.59742	0.85352	33.04199	0.5757
29.945	0:52:24	0.49301	60.73913	0.91489	32.73071	0.54038
30.583	0:52:26	0.49078	62.31509	0.8707	35.12461	0.57281
28.87	0:52:28	0.49231	58.64191	0.82533	34.97995	0.60305
27.774	0:52:30	0.50122	55.41279	0.91388	30.3913	0.55112
28.695	0:52:32	0.49596	57.85749	0.92734	30.94334	0.53688
41.644	0:52:34	0.49985	83.31299	0.76269	54.60148	0.69946
31.882	0:52:36	0.50248	63.44929	0.87062	36.61988	0.57848
31.56875		0.492776	64.06322	0.871216	36.45723	0.573488
	VO2/weigh	0.041885	36.23526			
	VCO/weigh	0.023691				
				MV/weigh	1.517728	64.06305

MV	Time	VCO2	MVb/VCO2	VO2	MV/VO2	RQ
73.534	1:58:08	0.23626	311.2419	0.17227	426.8532	1.6436
71.491	1:58:10	0.28007	255.2612	0.16511	432.9901	2.0709
69.592	1:58:12	0.36141	192.5569	0.22276	312.408	1.6581
74.692	1:58:14	0.40028	186.5994	0.20462	365.0279	2.1108
72.596	1:58:16	0.42509	170.778	0.35117	206.7261	1.2187
82.648	1:58:18	0.42277	195.4916	0.39124	211.2463	1.0973
75.519	1:58:20	0.44841	168.4151	0.34799	217.0149	1.3146
76.808	1:58:22	0.44266	173.5147	0.3761	204.2223	1.2156
78.665	1:58:24	0.4845	162.3633	0.34243	229.7258	1.5327
79.582	1:58:26	0.47221	168.531	0.45428	175.1827	1.0465
78.52	1:58:28	0.44903	174.8658	0.45606	172.1703	0.99822
77.541	1:58:30	0.46619	166.3292	0.44002	176.2215	1.0622

78.026	1:58:32	0.47543	164.1167	0.39914	195.4853	1.2236	
76.955	1:58:34	0.45245	170.0851	0.40124	191.7929	1.1514	
76.15493		0.415483	190.0107	0.337459	251.2191	1.38173	
		VO2/weigh	0.016224	225.6715			
		VCO/weigh	0.019975				
					MV/weigh	3.661295	183.2926

**G998**	**weight**	Time	MV	Time	VCO2	MVb/VCO2	VO2
	22.9	1:10:26	23.044	1:10:26	0.92891	24.80757	1.377
		1:10:28	26.37	1:10:28	0.90631	29.096	1.2954
		1:10:30	21.85	1:10:30	0.91633	23.84512	1.3488
		1:10:32	24.066	1:10:32	0.88987	27.0444	1.2992
		1:10:34	23.115	1:10:34	0.87551	26.40175	1.3182
		1:10:36	24.092	1:10:36	0.87031	27.68209	1.2757
		1:10:38	24.948	1:10:38	0.86866	28.7201	1.3043
		1:10:40	23.56	1:10:40	0.8538	27.59428	1.3948
		1:10:42	28.618	1:10:42	0.83445	34.29564	1.3284
		1:10:44	25.929	1:10:44	0.83531	31.04117	1.342
		1:10:46	26.322	1:10:46	0.82905	31.74959	1.3266
		1:10:48	23.737	1:10:48	0.8145	29.14303	1.3164
		1:10:50	23.907	1:10:50	0.81302	29.40518	1.2731
		1:10:52	25.07	1:10:52	0.80336	31.20643	1.1519
		1:10:54	24.953	1:10:54	0.81192	30.73332	1.2695
		1:10:56	23.943	1:10:56	0.80675	29.67834	1.1384
			24.59525		0.853629	28.90275	1.297481
				VO2/weigh	0.056659	18.95615	
	**weight**			VCO/weigh	0.037276		
	22.9						MV/weigh
		Time	MV	Time	VCO2	MVb/VCO2	VO2
		1:29:12	127.19	1:29:12	1.1678	108.9142	1.3973
		1:29:14	130.33	1:29:14	1.1484	113.4883	1.2712
		1:29:16	137.09	1:29:16	1.0844	126.4201	1.1511
		1:29:18	135.29	1:29:18	1.0905	124.0624	1.2666
		1:29:20	160.56	1:29:20	1.1465	140.0436	1.4456
		1:29:22	165.93	1:29:22	1.147	144.6643	1.448
		1:29:24	123.12	1:29:24	1.1965	102.9001	1.5564
		1:29:26	145.45	1:29:26	1.1834	122.9086	1.453
		1:29:28	128.79	1:29:28	1.1688	110.1899	1.4352
		1:29:30	130.27	1:29:30	1.2148	107.2358	1.4903
		1:29:32	134.66	1:29:32	1.2429	108.3434	1.4382
		1:29:34	174.34	1:29:34	1.2656	137.7528	1.4341
		1:29:36	173.34	1:29:36	1.2804	135.3796	1.4186
		1:29:38	142.85	1:29:38	1.2744	112.092	1.4504
		1:29:40	139.12	1:29:40	1.2669	109.8114	1.5261
			143.222		1.191887	120.2804	1.41214
				VO2/weigh	0.061666	101.422	

				VCO/weigh	0.052047		
							MV/weigh

**G810**	**weight**							
		16.7						
			Time	MV	Time	VCO2	MVb/VCO2	VO2
			0:21:12	36.066	0:21:12	0.66513	54.22399	1.1116
			0:21:14	35.957	0:21:14	0.66648	53.95061	1.0243
			0:21:16	36.738	0:21:16	0.66805	54.99289	1.1391
			0:21:18	37.16	0:21:18	0.66711	55.70296	1.1082
			0:21:20	44.428	0:21:20	0.66354	66.95602	1.095
			0:21:22	41.316	0:21:22	0.66953	61.70896	1.1148
			0:21:24	41.803	0:21:24	0.66615	62.75313	1.0774
			0:21:26	43.891	0:21:26	0.67894	64.64636	0.97888
			0:21:28	45.609	0:21:28	0.67342	67.72742	1.0405
			0:21:30	45.926	0:21:30	0.66591	68.96728	0.98169
			0:21:32	43.876	0:21:32	0.69729	62.9236	1.0835
			0:21:34	44.817	0:21:34	0.73271	61.16608	1.1186
			0:21:36	43.209	0:21:36	0.76917	56.17614	1.1675
			0:21:38	39.737	0:21:38	0.79968	49.69113	1.2206
			0:21:40	38.047	0:21:40	0.82439	46.1517	1.2427
			0:21:42	45.133	0:21:42	0.81868	55.12899	1.1283
				41.48206		0.707886	58.9292	1.102042
					VO2/weigh	0.065991	37.64109	
					VCO/weigh	0.042388		
	**weight**							MV/weigh
		16.7						
			Time	MV	Time	VCO2	MVb/VCO2	VO2
			1:21:12	58.287	1:21:12	0.81992	71.08864	0.77169
			1:21:14	62.726	1:21:14	0.87245	71.89638	0.57151
			1:21:16	61.03	1:21:16	0.88943	68.61698	0.71204
			1:21:18	60.188	1:21:18	0.86163	69.85365	0.67705
			1:21:20	59.805	1:21:20	0.8839	67.66037	0.58322
			1:21:22	57.249	1:21:22	0.88967	64.34858	0.65145
			1:21:24	59.237	1:21:24	0.893	66.33483	0.65512
			1:21:26	63.972	1:21:26	0.90544	70.65294	0.67418
			1:21:28	62.255	1:21:28	0.88991	69.95651	0.56818
			1:21:30	62.488	1:21:30	0.88476	70.62706	0.63592

1:21:32	61.743	1:21:32	0.89372	69.0854	0.6876
1:21:34	60.611	1:21:34	0.88241	68.68802	0.62377
1:21:36	62.473	1:21:36	0.90237	69.23213	0.70392
1:21:38	66.951	1:21:38	0.91003	73.5701	0.68612
1:21:40	62.901	1:21:40	0.87592	71.81135	0.68854
	61.46107		0.883637	69.56153	0.659354
			VO2/weigh	0.039482	93.21407
			VCO/weigh	0.052912	

MV/VO2	RQ
16.73493	0.67457
20.35665	0.70394
16.19958	0.68054
18.52371	0.68592
17.53528	0.66666
18.88532	0.68312
19.1275	0.67081
16.89131	0.6172
21.54321	0.63187
19.32116	0.62668
19.8417	0.62681
18.03175	0.62023
18.77857	0.64245
21.76404	0.70112
19.65577	0.63986
21.03215	0.7115
19.01391	0.661455

1.074028	28.81258
MV/VO2	RQ
91.02555	0.83782
102.5252	0.90368
119.0948	0.94651
106.8135	0.86122
111.0681	0.79474
114.5925	0.79437
79.10563	0.77026
100.1032	0.81759
89.73662	0.81677
87.41193	0.81788
93.63093	0.8658
121.5675	0.89743
122.1909	0.90523
98.49007	0.88138
91.16047	0.83267
101.9011	0.849557

6.254236	120.1641

MV/VO2	RQ
32.44512	0.60541
35.10397	0.65567
32.25178	0.58845
33.53185	0.60761
40.57352	0.61253
37.06136	0.61497
38.79989	0.62053
44.83798	0.69389
43.83373	0.65104
46.78259	0.68019
40.49469	0.64629
40.06526	0.66089
37.00985	0.66034
32.5553	0.66362
30.6164	0.66646
40.00089	0.72556
37.87276	0.647091

2.483956	58.5999

MV/VO2	RQ
75.53163	1.0696
109.7549	1.5811
85.71148	1.2636
88.89742	1.28
102.5428	1.5436
87.87935	1.3778
90.4216	1.3714
94.88861	1.3446
109.5692	1.5737
98.26393	1.4084

89.79494	1.3122	
97.16883	1.4233	
88.75014	1.2929	
97.57914	1.3502	
91.35417	1.293	
93.87387	1.365693	
		69.55463
MV/weigh	3.680303	

f(BPM)

WT 10 wee	RA 01	HO/HC
G1168 11 v	192.4825	351.2102
G1169 11w	166.3446	395.7097
G998 10 w	185.7971	388.8527

mdx/utrn	RA 01	HO/HC
G1214 10w	280.1532	365.1406
G1215 10w	328.0141	274.328
G810_10w	338.6793	415.9574

TVb(ml)

WT 10 wee	RA 01	HO/HC
G1168 11 v	0.242139	0.526484
G1169 11w	0.171769	0.462911
G998 10 w	0.141827	0.384773

mdx/utrn	RA 01	HO/HC
G1214 10w	0.128334	0.164658
G1215 10w	0.1059	0.121568
G810_10w	0.115914	0.149376

MVb(ml/min)

WT 10 wee	RA 01	HO/HC
G1168 11 v	46.62245	184.4391
G1169 11v	28.61148	182.3438
G998 10 w	26.12696	149.1114

mdx/utrn	RA 01	HO/HC
G1214 10v	35.67043	59.89632
G1215 10v	34.51562	33.26178
G810_10w	39.03189	61.83533

Penh

WT 10 wee	RA 01	HO/HC
G1168 11 v	1.839786	1.654496
G1169 11v	1.550283	1.314662
G998 10 w	1.08557	1.326313

mdx/utrn	RA 01	HO/HC
G1214 10v	1.945052	1.607106
G1215 10v	0.96739	1.728673
G810_10w	1.016092	1.25498

PIFb(ml/sec)

WT 10 wee	RA 01	HO/HC
G1168 11 \	3.533016	10.06997
G1169 11w	2.421247	10.05112
G998 10 w	2.127592	8.27172

mdx/utrn	RA 01	HO/HC
G1214 10w	2.435345	3.392903
G1215 10w	2.84133	2.15551
G810_10w	2.855392	3.558234

PEFb(ml/sec)

WT 10 wee	RA 01	HO/HC
G1168 11 \	3.743542	11.20372
G1169 11w	1.74975	10.13061
G998 10 w	1.466925	8.383121

mdx/utrn	RA 01	HO/HC
G1214 10w	2.282733	3.426047
G1215 10w	1.933925	1.910956
G810_10w	1.948964	3.267576

Date Sac	Mouse ID	Muscle	DOB	Group	Sex	Body Weight (g)	Order - day
25-Apr-24	M2392/G2106	DIA	11-Mar-24	dKO	M	15.8	1
21-May-24	M2405/G2231	DIA	6-Apr-24	dKO	M	16.6	1
21-May-24	M2406/G2232	DIA	6-Apr-24	dKO	M	19.4	2
21-May-24	M2407/G2233	DIA	6-Apr-24	dKO	M	18.5	3
12-Jun-24	M2424/G2342	DIA	28-Apr-24	dKO	M	11.1	1

Bundle Length (cm)	Bundle weight (g)	Bundle Length (mm)	Bundle weight (mg)	Bundle CSA (cm2)	Bundle CSA (mm2)
0.600	0.002	6	2	0.003174603	0.3175
0.729	0.0027	7.29	2.7	0.003527337	0.3527
0.544	0.0033	5.44	3.3	0.005777311	0.5777
0.622	0.0021	6.22	2.1	0.003215434	0.3215
0.662	0.0017	6.62	1.7	0.002445691	0.2446
0.631	0.002				

1Hz Force (mN)	15Hz Force (mN)	30Hz Force (mN)	50Hz Force (mN)	80Hz Force (mN)	120Hz Force (mN)
8.49559	9.179122	10.119433	19.445831	31.62561	36.116602
6.816576	7.769918	10.250503	18.040456	28.193055	31.903837
10.324352	13.456742	20.585898	34.691829	47.778891	53.986531
6.425496	7.719331	10.011824	18.77134	27.950483	33.037423
14.38669	15.85883	21.082646	31.835055	37.520674	40.274266
9.2897408	10.7967886	14.4100608	24.5569022	34.6137426	39.0637318

150Hz Force (mN)	300aHz Force (mN)	300bHz Force (mN)	1Hz Force (N)	15Hz Force (N)	30Hz Force (N)
36.686045	40.092036	37.959012	0.00849559	0.009179122	0.010119433
32.888269	36.294241	33.543979	0.006816576	0.007769918	0.010250503
55.417135	58.023512	57.111019	0.010324352	0.013456742	0.020585898
34.518645	38.84235	34.927844	0.006425496	0.007719331	0.010011824
40.700322	45.708809	41.148433	0.01438669	0.01585883	0.021082646
40.0420832	43.7921896	40.9380574			

50Hz Force (N)	80Hz Force (N)	120Hz Force (N)	150Hz Force (N)	300aHz Force (N)	300bHz Force (N)
0.019445831	0.03162561	0.036116602	0.036686045	0.040092036	0.037959012
0.018040456	0.028193055	0.031903837	0.032888269	0.036294241	0.033543979
0.034691829	0.047778891	0.053986531	0.055417135	0.058023512	0.057111019
0.01877134	0.027950483	0.033037423	0.034518645	0.03884235	0.034927844
0.031835055	0.037520674	0.040274266	0.040700322	0.045708809	0.041148433

1Hz Force (N/cm2)	15Hz Force (N/cm2)	30Hz Force (N/cm2)	50Hz Force (N/cm2)	80Hz Force (N/cm2)	120Hz Force (N/cm2)
2.67611085	2.89142343	3.187621395	6.125436765	9.96206715	11.37672963
1.932499296	2.202771753	2.906017601	5.114469276	7.992731093	9.04473779
1.787051473	2.329239706	3.563231799	6.00484022	8.270091679	9.344577729
1.998329256	2.400711941	3.113677264	5.83788674	8.692600213	10.27463855
5.882463658	6.484395725	8.620321903	13.01679219	15.34154147	16.46743665
2.855290907	3.261708511	4.278173992	7.219885039	10.05180632	11.30162407

150Hz Force (N/cm2)	300aHz Force (N/cm2)	300bHz Force (N/cm2)	1Hz Force (kN/m2)	15Hz Force (kN/m2)	30Hz Force (kN/m2)
11.55610418	12.62899134	11.95708878	26.8	28.9	31.9
9.323824262	10.28941732	9.509718047	19.3	22.0	29.1
9.592202276	10.04334244	9.885398198	17.9	23.3	35.6
10.7352986	12.07997085	10.86255948	20.0	24.0	31.1
16.64164342	18.68952537	16.8248681	58.8	64.8	86.2
11.56981455	12.74624947				
		**% loss**			
	0.67190256	0.053203185			
	0.779699277	0.075776815			
	0.157944243	0.015726263			
	1.217411366	0.10077933			
	1.864657269	0.099770178			
		0.069051154			

50Hz Force (kN/m2)	80Hz Force (kN/m2)	120Hz Force (kN/m2)	150Hz Force (kN/m2)	300aHz Force (kN/m2)	300bHz Force (kN/m2)
61.3	99.6	113.8	115.6	126.3	119.6
51.1	79.9	90.4	93.2	102.9	95.1
60.0	82.7	93.4	95.9	100.4	98.9
58.4	86.9	102.7	107.4	120.8	108.6
130.2	153.4	164.7	166.4	186.9	168.2

35% load	35% load	35% load	35% load	35% load	35% load
Afterloaded Baseline (mN)	Afterloaded Clamp Max (mN)	Afterloaded Clamp Min (mN)	Afterloaded Clamp Avg (mN)	Shortening Velocity (mm/s)	Shortening Velocity (Lo/s)
9.078721	24.5511	23.8701	24.2106	1.65E+01	2.76E+00
6.755726	21.503	20.6924	21.0977	1.65E+01	2.27E+00
6.754422	29.447401	28.4746	28.9610005	1.88E+01	3.45E+00
5.736902	21.7624	21.146301	21.4543505	1.55E+01	2.50E+00
6.936012	25.361698	24.5186	24.940149	1.63E+01	2.46E+00

35% load	35% load	35% load	35% load	35% load	35% load	35% load
Afterloaded active force (mN)	Afterloaded active force (N)	Afterloaded active force (N/cm2)	Afterloaded active force (%Max)	Power (W;N*m/s)	Power (W/kg)	Power (N/cm2*Lo/s)
15.1	0.015	4.767	37.7	0.00025028	125.1	13.140
14.3	0.014	4.066	39.5	0.00023707	87.8	9.219
22.2	0.022	3.844	38.3	0.00041637	126.2	13.248
15.7	0.016	4.888	40.5	0.00024425	116.3	12.212
18.0	0.018	7.362	39.4	0.00029275	172.2	18.081
					125.5	

15% load	15% load	15% load	15% load	15% load
**Afterloaded Baseline (mN)**	**Afterloaded Clamp Max (mN)**	**Afterloaded Clamp Min (mN)**	**Afterloaded Clamp Avg (mN)**	**Shortening Velocity (mm/s)**
			0	
			0	
			0	
			0	
			0	

15% load	15% load	15% load	15% load	60% load	60% load	60% load
Afterloaded active force (mN)	Afterloaded active force (%Max)	Power (W;N*m/s)	Power (W/kg)	Afterloaded Baseline (mN)	Afterloaded Clamp Max (mN)	Afterloaded Clamp Min (mN)
0.0	0.0	0	0.0			
0.0	0.0	0	0.0			
0.0	0.0	0	0.0			
0.0	0.0	0	0.0			
0.0	0.0	0	0.0			

60% load<br>Afterloaded Clamp Avg (mN)	60% load<br>Shortening Velocity (mm/s)	60% load<br>Afterloaded active force (mN)	60% load<br>Afterloaded active force (%Max)	60% load<br>Power (W;N*m/s)	60% load<br>Power (W/kg)	25% load<br>Afterloaded Baseline (mN)
0		0.0	0.0	0	0.0	
0		0.0	0.0	0	0.0	
0		0.0	0.0	0	0.0	
0		0.0	0.0	0	0.0	
0		0.0	0.0	0	0.0	

25% load<br>Afterloaded Clamp Max (mN)	25% load<br>Afterloaded Clamp Min (mN)	25% load<br>Afterloaded Clamp Avg (mN)	25% load<br>Shortening Velocity (mm/s)	25% load<br>Afterloaded active force (mN)	25% load<br>Afterloaded active force (%Max)	25% load<br>Power (W;N*m/s)
		0		0.0	0.0	0
		0		0.0	0.0	0
		0		0.0	0.0	0
		0		0.0	0.0	0
		0		0.0	0.0	0

25% load	75% load	75% load	75% load	75% load	75% load
Power (W/kg)	Afterloaded Baseline (mN)	Afterloaded Clamp Max (mN)	Afterloaded Clamp Min (mN)	Afterloaded Clamp Avg (mN)	Shortening Velocity (mm/s)
0.0				0	
0.0				0	
0.0				0	
0.0				0	
0.0				0	

75% load	75% load	75% load	75% load
Afterloaded active force (mN)	Afterloaded active force (%Max)	Power (W;N*m/s)	Power (W/kg)
0.0	0.0	0	0.0
0.0	0.0	0	0.0
0.0	0.0	0	0.0
0.0	0.0	0	0.0
0.0	0.0	0	0.0
